# HDL cholesterol levels and susceptibility to COVID-19

**DOI:** 10.1016/j.ebiom.2022.104166

**Published:** 2022-07-15

**Authors:** Vignesh Chidambaram, Amudha Kumar, Marie Gilbert Majella, Bhavna Seth, Ranjith Kumar Sivakumar, Dinesh Voruganti, Mahesh Bavineni, Ahmad Baghal, Kim Gates, Annu Kumari, Subhi J. Al'Aref, Panagis Galiatsatos, Petros C. Karakousis, Jawahar L. Mehta

**Affiliations:** a Department of Internal Medicine, University of Arkansas for Medical Sciences, AR, USA; b Department of Community Medicine, Sri Venkateshwaraa Medical College Hospital & Research Centre, Pondicherry, India; c Division of Pulmonary and Critical Care Medicine, Department of Medicine, Johns Hopkins School of Medicine, Baltimore, MD, USA; d Department of Anaesthesia and Intensive Care, The Chinese University of Hong Kong, Prince of Wales Hospital, Shatin, Hong Kong, China; e Division of Cardiovascular Medicine, University of Arkansas for Medical Sciences, Little Rock, AR, USA; f Department of Biomedical Informatics, University of Arkansas for Medical Sciences, Little Rock, AR, USA; g Division of Infectious Diseases, Department of Medicine, Johns Hopkins School of Medicine, Baltimore, MD, USA; h Department of International Health, Johns Hopkins Bloomberg School of Public Health, Baltimore, MD, USA; i Division of Cardiovascular Medicine, Central Arkansas Veterans Healthcare System, Little Rock, AR, USA

**Keywords:** SARS-CoV-2, Risk, LDL, Total cholesterol, Triglycerides

## Abstract

**Background:**

Host cell-membrane cholesterol, an important player in viral infections, is in constant interaction with serum high-density lipoprotein-cholesterol (HDL-C) and low-density lipoprotein-cholesterol (LDL-C). Low serum lipid levels during hospital admission are associated with COVID-19 severity. However, the effect of antecedent serum lipid levels on SARS-CoV-2 infection risk has not been explored.

**Methods:**

From our retrospective cohort from the Arkansas Clinical Data-Repository, we used log-binomial regression to assess the risk of SARS-CoV-2 infection among the trajectories of lipid levels during the 2 years antecedent to COVID-19 testing, identified using group-based-trajectory modelling. We used mixed-effects linear regression to assess the serum lipid level trends followed up to the time of, and 2-months following COVID-19 testing.

**Findings:**

Among the 11001 individuals with a median age of 59 years (IQR 46-70), 1340 (12.2%) tested positive for COVID-19. The highest trajectory for antecedent serum HDL-C was associated with the lowest SARS-CoV-2 infection risk (RR 0.63, 95%CI 0.46-0.86). Antecedent serum LDL-C, total cholesterol (TC), and triglycerides (TG) were not independently associated with SARS-CoV-2 infection risk. In COVID-19 patients, serum HDL-C (-7.7, 95%CI -9.8 to -5.5 mg/dL), and LDL-C (-6.29, 95%CI -12.2 to -0.37 mg/dL), but not TG levels, decreased transiently at the time of testing.

**Interpretation:**

Higher antecedent serum HDL-C, but not LDL-C, TC, or TG, levels were associated with a lower SARS-CoV-2 infection risk. Serum HDL-C, and LDL-C levels declined transiently at the time of infection. Further studies are needed to determine the potential role of lipid-modulating therapies in the prevention and management of COVID-19.

**Funding:**

Research reported in this publication was supported by the National Center for Advancing Translational Sciences of the National Institutes of Health under Award Number UL1 TR003107.

**Research in context**

**Evidence before this study**

It is well known that elevated serum low-density lipoprotein-cholesterol (LDL-C) and low high-density lipoprotein-cholesterol (HDL-C) increase the risk of cardiovascular disorders. On the contrary, recent evidence suggests that low serum levels of LDL-C, HDL-C, and total cholesterol (TC) at hospital admission are associated with severe disease and mortality in COVID-19.^7^^,^^8^ However, these associations were based on measurements made during hospital admission and suffer from the possibility of reverse causality due to the acute inflammatory response in COVID-19. Additionally, clinical data on the impact of past HDL-C and LDL-C levels on the risk and outcome of infectious diseases remain inconsistent.^13^^,^^14^

**Added value of this study**

From our retrospective cohort of 11,001 individuals, we demonstrate that patients with higher antecedent HDL-C levels have a lower risk of SARS-CoV-2 infection. The risk is the lowest in the subgroup with higher levels of HDL-C and lower levels of LDL-C. Additionally we show that antecedent LDL-C, and TC are not independently associated with the risk of SARS-CoV-2 infection, and the drop in these lipid fractions is transient with a return to pre-infection levels by 2 months following infection.

**Implications of all the available evidence**

Our findings have important clinical applications due to a potential causal relationship between low HDL-C levels and susceptibility to SARS-CoV-2 infection. The results of our study could provide the impetus for large-scale clinical trials using lipid modulating drugs such as statins and CETP inhibitors, aimed at increasing HDL-C levels in the prevention and amelioration of SARS-CoV-2 infection or infections in general.

Alt-text: Unlabelled box

## Introduction

The burden of Coronavirus disease-2019 (COVID-19) continues to remain high worldwide^1^ and is currently the most common cause of death due to a single infectious agent.^2^ There is a persistent need to understand the host factors that can lead to increased susceptibility and adverse outcomes in COVID-19 patients in order to develop novel pharmacological interventions for prevention and/or treatment.

Patients with comorbidities, such as hypertension (HTN), diabetes mellitus (DM), and pre-existing cardiovascular diseases have poor COVID-19-related outcomes.^3^^,^^4^ It is well known that elevated serum low-density lipoprotein-cholesterol (LDL-C) and low high-density lipoprotein-cholesterol (HDL-C) increase the risk of cardiovascular disorders.^5^^,^^6^ On the contrary, recent evidence suggests that low serum levels of LDL-C, HDL-C, and total cholesterol (TC) at hospital admission are associated with severe disease and mortality in COVID-19.^7^^,^^8^ However, these associations were based on measurements made during hospital admission and suffer from the possibility of reverse causality due to the acute inflammatory response in COVID-19.^9^

Among the various serum lipoprotein fractions, pre-clinical evidence suggests that HDL-C is an important modulator of inflammation,^10^^,^^11^ with potential mechanisms including interference with viral fusion, reduction in the rate of bacterial complications, and neutralization of exaggerated immune responses.^12^ However, clinical data on the impact of past HDL-C and LDL-C levels on the risk and outcome of infectious diseases remain inconsistent.^13^^,^^14^ Understanding the association of antecedent lipid levels with SARS-CoV-2 infection risk is important as serum lipids are attractive targets due to the ready availability of serum lipid measurements and the pharmacological agents, such as statins, that can modify them.^15^ Studies using the United Kingdom (UK) biobank data^16^ attempted to assess this association, but the lipid measurements were performed over ten years ago and might not accurately represent current values.

To further characterize this temporal association, we carried out a retrospective cohort study to assess the association of lipid levels assayed during the two years antecedent to COVID-19 testing, with the risk of SARS-CoV-2 infection, need of hospitalization, disease severity, and mortality using group-based trajectory modelling and the tertile approach. We also evaluated the effect of each lipid fraction across various levels of the other lipid fractions. Additionally, we determined the trends of available lipid levels before, at the time of, and two months following testing for SARS-CoV-2 infection.

## Methods

### Design and setting

Our retrospective cohort study included individuals ≥ 18 years of age who were tested for COVID-19 in any of the participating centres which provide data to the Arkansas Clinical Data Repository (AR-CDR). This repository contains deidentified real-time patient data from electronic medical records. We included all patients with one or more of the following serum lipid levels tested in the two years antecedent to their index COVID-19 testing: LDL-C, HDL-C, TC, or TG (triglycerides). We divided the two years antecedent to COVID-19 testing into eight 3-month time periods and only included participants tested for the above lipid levels in at least two of the eight 3-month periods. We excluded subjects who only had lipid levels tested within two weeks of testing for COVID-19 to account for the estimated incubation period for COVID-19.

### Patient characteristics

Data collected included information on patient characteristics, such as age, sex, race, body mass index (BMI), and comorbidities, including DM, HTN, coronary artery disease, heart failure, solid organ or bone marrow transplantation, chronic kidney disease, chronic obstructive pulmonary disease, HIV, tobacco use, and alcohol use disorder. The Charlson comorbidity index (CCI) and Elixhauser score were calculated from the available variables.^17^ Prior COVID-19 vaccinations received by the patient were recorded at the time of index testing. Laboratory data on renal and liver function during the last 2 years were obtained from the database.

### Exposures

The exposures assessed in our study were serum lipids, namely LDL-C, HDL-C, TC, and TG. Two approaches were used to determine the level of exposure based on lipid levels available at various time points in the 2 years antecedent to COVID-19 testing. In the first, individuals were categorized based on the trajectories of lipid levels for each of the above-mentioned lipids using group-based trajectory modelling (GBTM) using the “traj” plugin in STATA.^18^^,^^19^ The GBTM identified mutually exclusive clusters of individuals, through the semiparametric method, and assigns them to 1 of the 3 categories of low, medium, and high lipid levels based on their longitudinal trajectory over at least two of the eight 3-month time periods. In the second approach, we categorized patients based on tertiles (low, medium, and high) of the weighted mean of the available lipid levels for each of the six months during the last 2 years. Low, medium, and high trajectories or tertiles are represented by numbers 1,2, and 3, respectively. Lipid levels “at the time of testing” were defined as levels assessed anytime within 14 days before or after COVID-19 testing.

### Outcomes

#### SARS-CoV2 infection testing

The primary outcome was a positive test for SARS-CoV-2 infection, and this was established by quantitative reverse‐transcription polymerase chain (RT-PCR)-based nucleic acid amplification (NAAT) test techniques for SARS-CoV-2 RNA using a nasal swab sample, at any of our collaborating centres in AR-CDR. We included information about all available tests for each patient. For patients with multiple tests, patients who remained negative on all tests were considered negative, and the first negative test was considered the index test. If the patients were positive on at least one of the tests, these patients were considered positive, and the first positive test was considered the index test.

#### Admission and in-hospital outcomes

Other primary outcomes measured in our study were the requirement for hospital admission among COVID-19 positive patients, and the in-hospital development of severe disease and mortality. Patients were classified as having severe COVID-19 if one of the following features was present: hypoxia (defined by SpO2 <94% on room air with PaO_2_/FiO_2_ < 300 mm Hg or respiratory rate > 30/min); lung infiltrates > 50% of lung volume on chest imaging; septic shock; or multiple organ dysfunction.^20^ Other in-hospital outcomes assessed are described in the supplementary document.

#### Inflammatory markers following COVID-19 testing

Serum inflammatory markers, such as C-reactive protein (CRP), erythrocyte sedimentation rate (ESR), serum IL-6, and procalcitonin at all available timepoints in the first 60 days following the positive COVID-19 test were noted. Repeated measurements were tracked longitudinally and compared among the lipid trajectories and tertiles.

### Statistical analysis

We compared differences in demographic and clinical characteristics stratified by the three trajectories and tertiles of LDL-C, HDL-C, TC, and TG, in the two years antecedent to COVID-19 testing using ANOVA for normally distributed data and Kruskal–Wallis test for non-normally distributed data, and Chi-square test for categorical variables. The normality of the data was assessed by Shapiro–Wilk test. Comparison of binary outcomes between different trajectories and tertiles was performed using Chi-square test. We then tested the association of the trajectories and tertiles of past lipid levels to the risk of testing positive for COVID-19, admission following SARS-CoV-2 infection, and COVID-19 severity using log-binomial regression. Kaplan-Meier and Cox-proportional hazards methods were used to measure the association between the lipid trajectories and tertiles with in-hospital mortality among patients admitted to the hospital for SARS-CoV-2 infection. We used the lowest trajectory/tertile (i.e., trajectory/tertile 1) as the reference for all the above analyses. A 2-tailed p-value of < 0.05 was considered statistically significant. Covariates for statistical adjustment were chosen *a priori* based on known association with lipid levels and risk of infectious disease. We calculated adjusted effect sizes based on 2 models: Model 1 adjusted for age, gender, race, CCI, intensity of statin therapy, calcium channel blocker use, angiotensin converting enzyme inhibitor use, alcohol intake and the number of times the patient was tested for COVID-19; Model 2 adjusted for all the variables in the model 1 along with DM, HTN, HIV, BMI and COVID-19 vaccination, as specified in the footnotes of Tables.

We used mixed-effects linear regression analysis to evaluate the trends in lipid levels of patients followed up to the time of, and 60 days after SARS-CoV-2 infection testing, after adjusting for the interaction between lipid tertile/trajectory and time of testing, with a patient-level random intercept. We also analysed the association of antecedent serum lipid levels with the longitudinal data of available inflammatory markers, namely CRP, ESR, IL-6, and procalcitonin, in the first 60 days after COVID-19 diagnosis using mixed-effects linear regression analyses. If the inflammatory markers were missing, they were considered to be missing at random. For all missing information, multiple imputation was performed using a chained equation approach^21^ with 40 sets of imputations was used for each missing value. We performed sensitivity analyses using mixed-effects Poisson regression analysis with robust error-variance to assess the risk of COVID-19 across different trajectories/tertiles of antecedent lipid levels, taking into consideration all available COVID-19 tests for each patient. We also conducted sensitivity analysis to assess the risk of COVID-19 positivity by considering mean antecedent lipid levels of each patient in the past 2 years as continuous variables. Additional sensitivity analysis was conducted to assess the risk of COVID-19 positivity after adjusting for the setting of COVID-19 testing, i.e., either asymptomatic screening either as contact screening or testing prior to a procedure or testing due to symptoms suggestive of SARS-CoV-2 infection. Also, we performed the analysis of antecedent lipid levels with risk of infection after dividing the individuals in the study into subgroups according to low, medium, and high levels of HDL-C with low, medium, and high levels of LDL-C or TG in the trajectory and tertile models. All analyses were performed using Stata, version 16.0 IC (StataCorp LP, College Station, TX).^22^

### Ethics

The study team used de-identified data curated by the AR-CDR (Arkansas Clinical Data Repository) and did not have access to Protected Health Information (PHI). The study was classified as “non-human subjects research” by the IRB at the University of Arkansas for Medical Sciences (UAMS), Little Rock, Arkansas (IRB Number: 263352).

### Role of Funders

The funders had no role in the study design, data collection, data analyses, interpretation, or writing of report.

## Results

### Study population, sociodemographic characteristics, and comorbidities

**Figure 1:**
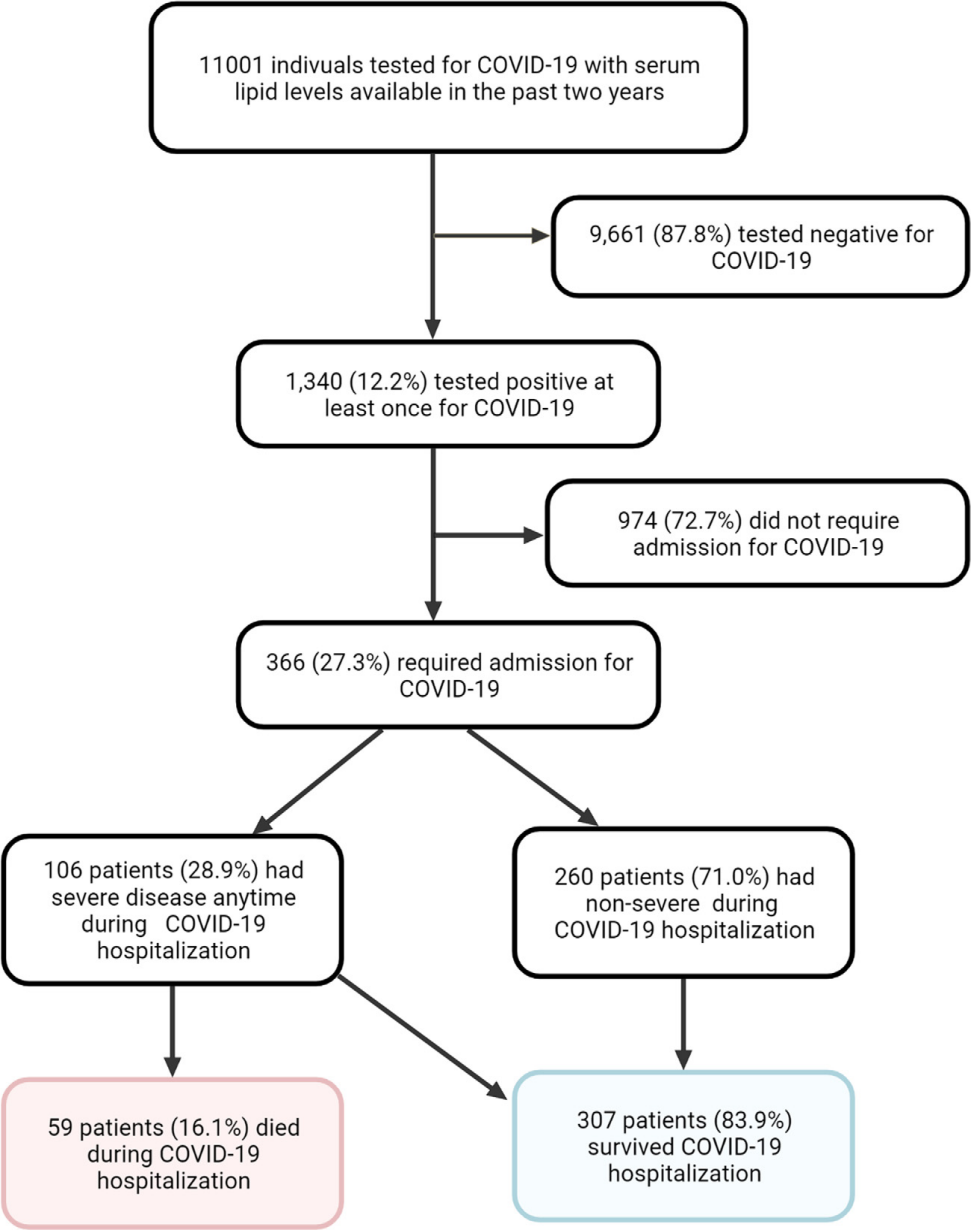
**Flow chart of the individuals included in the study**.

11,001 individuals with COVID-19 testing and lipid levels measured in the last 2 years were included in our study (Figure 1). Among them, 5389 individuals (54.7%) received asymptomatic screening, and the remaining individuals were tested due to the presence of COVID-19 symptoms. The median age was 59 (IQR: 46–70, range: 18–89) years and 4486 individuals (40.8%) were males. Our cohort consisted of 53.4% White, 40.1% African American and 3.5% Hispanic population, and the rest were native Americans and Asians. The flow chart of the study cohort and inclusion is presented in Figure 1. The demographic characteristics of the patients, use of cardiovascular medications, and laboratory data on renal and liver function in the last two years are presented in Supplementary Tables 1A and 1B.

### Stratification based on lipid tertiles and trajectories

**Figure 2:**
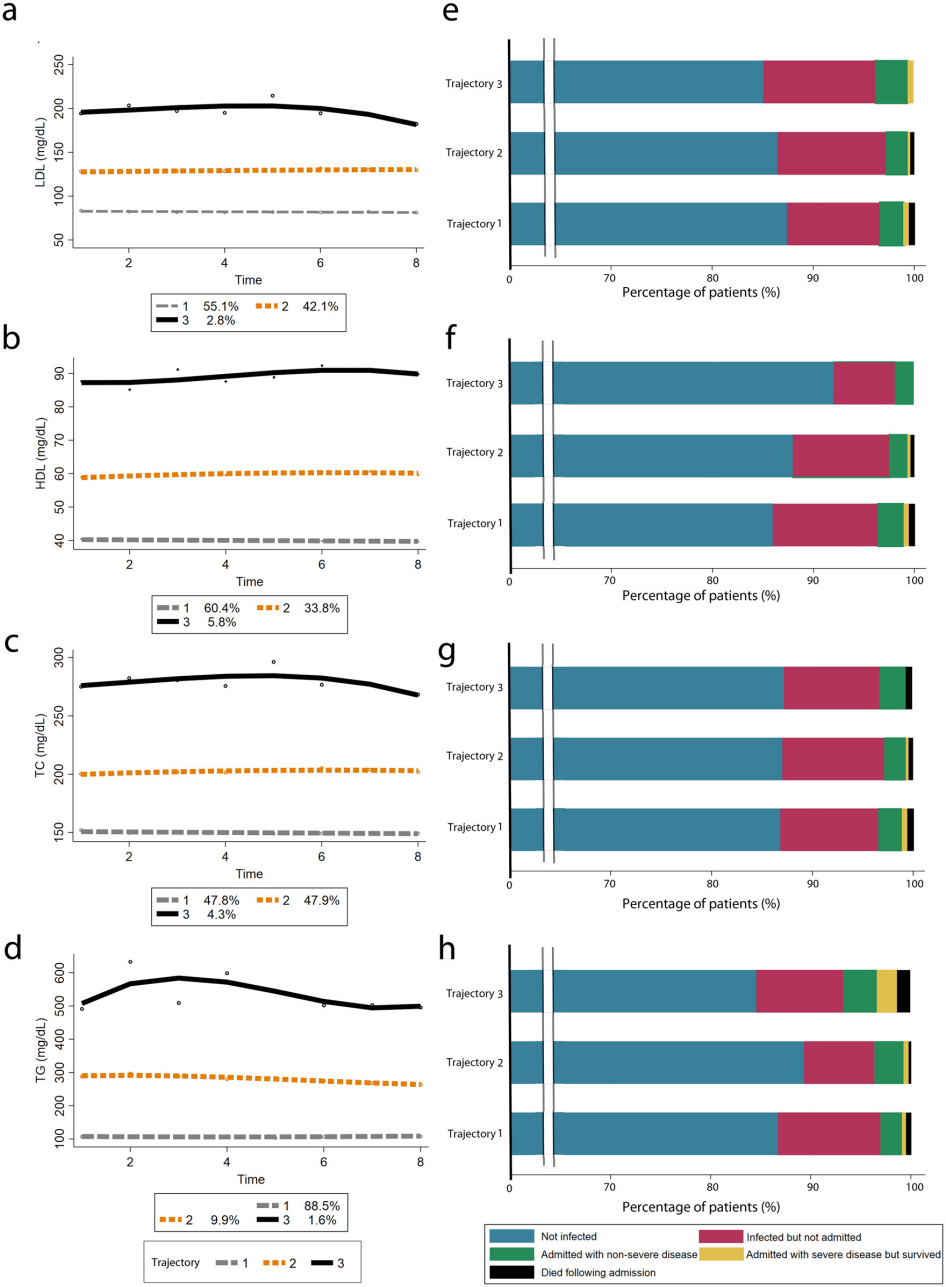
**Trajectories of LDL-C, HDL-C, TC and TG levels during the two years antecedent to COVID-19 testing using group-based trajectory modelling (GBTM) and COVID-19 related outcomes**. The *first row* (a, e) corresponds to LDL-C; *second row* (b, f) to HDL-C; *third row* (c, g) to TC, and *fourth row* (d, h) to TG. Low, medium, and high trajectories are represented by numbers 1,2, and 3, respectively. The *left panel* (a-d) presents the trajectories of lipid levels during the past two years over the eight 3-month time periods before COVID-19 testing (X-axis). The *right panel* (e-h) presents the outcomes in patients in each of the trajectories: the five outcomes represented are i) not infected (COVID-19 negative), ii) infected (COVID-19 positive) but not requiring admission, iii) admitted with non-severe COVID-19, iv) admitted with severe COVID-19 but survived and v) in-hospital death. Patients in the trajectories 2 and 3 of HDL-C had higher proportion of patients with negative COVID-19 tests.

The levels of lipids in each of the trajectories and tertiles are shown in Supplementary Tables 2 and 3. Based on the group-based trajectory modelling (GBTM), the trajectories 1, 2, and 3 of LDL-C had median (range) values of 82 (7-130), 132 (82-215) and 202.5 (155-242) mg/dL. The trajectories 1, 2, and 3 of HDL-C had median (range) values of 40.5 (14-52), 60.7 (50.5-83.1) and 88(76.4-148) mg/dL, respectively. The trajectories 1, 2, and 3 of TC had median (range) values of 148 (51-194.5), 204 (144.5-277) and 286 (242.5-636) mg/dL respectively. The trajectories 1, 2, and 3 of TG had median (range) values of 97.0 (13.0-711.5), 278.4 (189.5-729.0) and 515.9 (391.0-1453.5) mg/dL respectively. The lipid trajectories over the 2-year time period are presented in Figure 2.

The tertiles 1, 2, and 3 of LDL-C had median (range) values of 70 (7-87), 103 (87.3-119) and 140 (119.3-242) mg/dL, respectively. The tertiles 1, 2, and 3 of HDL-C had median (range) values of 35 (5-41.5), 47 (41.6-54) and 64.5(54.3-148) mg/dL, respectively. The tertiles 1, 2, and 3 of TC had median (range) values of 139 (29-159), 179 (159.3-197) and 222 (197.5-636) mg/dL, respectively. The trajectories 1, 2, and 3 of TG had median (range) values of 61 (13-82), 105.5 (82.2-137) and 193 (137-1491) mg/dL, respectively.

Patient characteristics stratified by trajectories of antecedent serum lipid levels are shown in Supplementary Tables 1A and 1B.

### Outcomes

Among 11001 patients tested for SARS-CoV-2 infection, 1340 (12.2%) tested positive at least once. The median number of times each patient was tested was once (IQR 1-2). Among the patients testing positive for SARS-CoV-2 infection, 366 patients (27.3%) were admitted due to SARS-CoV-2 infection with a median duration of hospital stay of 6 days (IQR 3-12). Eighty-six patients (23.5%) had severe COVID-19 at the time of hospital admission, with 20 more patients (5.6%) developing severe COVID-19 during the hospital stay. Among the 366 admitted patients, 59 patients (16.7%) died during the hospital stay. The level of oxygen support required and the need for ECMO, vasopressor support and renal replacement are described in Tables 1A, 1B and supplementary tables 4A and 4B.

**Table 1A:** Patient outcomes based on the trajectories of LDL-C and HDL-C during the 2 years antecedent to COVID-19 testing.

Patient outcomes	Unit	Total (N = 11001)	LDL-C			p-value	HDL-C			p-value
			Trajectory 1	Trajectory 2	Trajectory 3		Trajectory 1	Trajectory 2	Trajectory 3	
Number of COVID-19 tests done	Median (IQR)	1 (1-2)	1 (1-3)	1 (1-2)	2 (1-3)	**0.001**	2 (1-3)	1 (1-2)	1 (1-2)	**<0.001**
Asymptomatic screening (%)	n (%)	5389 (54.7%)	2450 (52.9%)	1755 (54.0%)	85 (49.4%)	**0.368**	2613 (52.0%)	1469 (55.5%)	237 (53.4%)	**0.016**
Number of patients testing positive at least once for COVID-19	n (%)	1340 (12.2%)	655 (12.7%)	502 (13.5%)	28 (14.8%)	0.371	801 (14.08%)	357 (12.01%)	39 (7.88%)	**<0.001**
COVID-19 outcomes										
Admission for COVID-19 (%)	n (%)	366 (27.3%)	177 (27.0%)	103 (20.5%)	7 (25.0%)	**0.038**	209 (26.09%)	73 (20.45%)	9 (23.08%)	0.116
Duration of hospital admission (days)	Median (IQR)	6 (3-12)	6 (4-13)	4 (2-9)	4 (3-5)	0.086	6(3-12)	5 (4-13)	2 (2-5)	0.116
Severe COVID-19 (%) at admission	n (%)	86/366 (23.5%)	48/184 (26.1%)	20/106 (18.9%)	1/7 (14.3%)	0.319	52/216 (24.07%)	17/76 (22.37%)	1/8 (11.11%)	0.651
Severe COVID-19 (%) anytime during hospitalization	n (%)	106/366 (28.9%)	60/184 (32.6%)	25/106 (29.0%)	1/7 (14.3%)	0.182	65/216 (30.09%)	21/76 (27.63%)	1/8 (11.11%)	0.450
Morality due to COVID-19 (%)	n (%)	59/366 (16.1%)	34/184 (18.5%)	16/106 (15.1%)	0/7 (0%)	0.368	36/216 (16.67%)	12/76 (15.79%)	1/8 (11.11%)	0.899
Highest oxygen support required - None - Nasal cannula - High flow oxygen - NIV - Intubation	n (%)	115 (31.4%) 143 (39.2%) 44 (12.1%) 13 (3.6%) 51 (13.9%)	56 (30.4%) 66 (35.9%) 22 (11.9%) 9 (4.9%) 31 (16.9%)	36 (33.9%) 45 (42.5%) 10 (9.4%) 3 (2.8%) 12 (11.3%)	3 (42.9%) 2 (28.6%) 2 (28.6%) 0 (0%) 0 (0%)	0.523	67 (31.0%) 82 (38.0%) 22 (10.2%) 10 (4.6%) 35 (16.2%)	27 (35.5%) 27 (35.5%) 12 (15.8%) 3 (3.9%) 7 (9.2%)	2 (22.2%) 6 (66.7%) 0 (28.6%) 0 (0%) 1 (11.1%)	0.459
ECMO	n (%)	1 (0.3%)	1 (0.6%)	0 (0%)	0 (0%)	0.734	1 (0.5%)	0 (0%)	0 (0%)	0.820
Vasopressor use	n (%)	36 (9.9%)	22 (12.0%)	7 (6.6%)	0 (0%)	0.222	25 (11.6%)	3 (3.9%)	0 (0%)	0.088
Renal replacement	n (%)	42 (11.5%)	28 (15.3%)	8 (7.6%)	0 (0%)	0.092	29 (13.5%)	7 (9.2%)	0 (0%)	0.327

COVID-19, Coronavirus disease-2019; ECMO, Extracorporeal Membrane oxygenation; IQR, interquartile range; LDL-C, low density lipoprotein cholesterol; HDL-C, high density lipoprotein cholesterol; NIV, Non-invasive ventilation.

**Table 1B:** Patient outcomes based on the trajectories of TC and TG during the 2 years antecedent to COVID-19 testing.

Patient outcomes	Unit	Total (N = 11001)	TC			p-value	TG			p-value
			Trajectory 1	Trajectory 2	Trajectory 3		Trajectory 1	Trajectory 2	Trajectory 3	
Number of COVID-19 tests done	Median (IQR)	1 (1-2)	2 (1-3)	1 (1-2)	2 (1-3)	**<0.001**	1 (1-3)	1 (1-3)	2 (1-3)	**0.012**
Asymptomatic screening (%)	n (%)	5389 (54.7%)	2052 (52.7%)	2148 (54.1%)	131 (48.3%)	0.115	3869 (53.3%)	393 (54.1%)	66 (48.2%)	**0.016**
Number of patients testing positive at least once for COVID-19	n (%)	1340 (12.2%)	578 (13.2%)	583 (12.9%)	39 (12.7%)	0.918	1087 (13.3%)	88 (10.6%)	23 (15.3%)	0.070
COVID-19 outcomes										
Admission for COVID-19 (%)	n (%)	366 (27.3%)	154 (26.6%)	128 (21.9%)	10 (25.6%)	0.174	252 (23.2%)	30 (34.1%)	10 (43.5%)	0.007
Duration of hospital admission (days)	Median (IQR)	6 (3-12)	7 (3-13)	5 (2-10)	5 (3-8)	0.471	6 (3-12)	6 (3-12)	6 (2-13)	0.482
Severe COVID-19 (%) at admission	n (%)	86/366 (23.5%)	41/158 (25.9%)	28/134 (20.9%)	2/10 (20%)	0.577	60/260 (23.1%)	6/32 (18.8%)	5/10 (50%)	0.115
Severe COVID-19 (%) anytime during hospitalization	n (%)	106/366 (28.9%)	53/158 (33.5%)	33/134 (24.6%)	2/10 (20%)	0.201	77/260 (29.6%)	6/32 (18.8%)	5/10 (50%)	0.149
Morality due to COVID-19 (%)	n (%)	59/366 (16.1%)	29/158 (18.4%)	21/134 (15.7%)	0/10 (0%)	0.297	46/260 (17.7%)	2/32 (6.3%)	2/10 (20%)	0.248
Highest oxygen support required - None - Nasal cannula - High flow oxygen - NIV - Intubation	n (%)	115 (31.4%) 143 (39.2%) 44 (12.1%) 13 (3.6%) 51 (13.9%)	47 (29.8%) 56 (35.4%) 21 (13.3%) 8 (5.1%) 26 (16.5%)	45 (33.6%) 56 (47.8%) 12 (8.9%) 4 (2.9%) 17 (12.7%)	4 (40%) 3 (30%) 2 (20%) 1 (10%) 0 (0%)	0.536	79 (30.4%) 101 (38.9%) 29 (11.2%) 11 (4.2%) 40 (15.4%)	14 (43.8%) 12 (37.5%) 4 (12.5%) 1 (3.2%) 1 (3.2%)	3 (30%) 2 (20%) 2 (20%) 1 (10%) 2 (20%)	0.505
ECMO	n (%)	1 (0.3%)	0 (0%)	1 (0.8%)	0 (0%)	0.535	1 (0.4)	0 (0%)	0 (0%)	0.922
Vasopressor use	n (%)	36 (9.9%)	17 (10.8%)	12 (8.9%)	0 (0%)	0.498	23 (8.9%)	4 (12.5%)	2 (20%)	0.426
Renal replacement	n (%)	42 (11.5%)	22 (14.0%)	13 (9.7%)	1 (10%)	0.518	28 (10.8%)	5 (15.6%)	3 (30%)	0.148

COVID-19, Coronavirus disease-2019; ECMO, Extracorporeal Membrane oxygenation; IQR, interquartile range; TC, Total Cholesterol; TG, Triglycerides; NIV, Non-invasive ventilation.

### Risk of testing positive for SARS-CoV-2 infection

**Figure 3:**
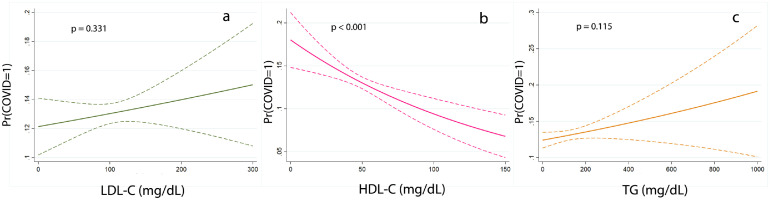
**Coefficient plot for the association of antecedent lipid levels with the probability of testing positive for COVID-19**. There is an inverse relationship between antecedent HDL-C levels and the probability of testing positive for COVID-19 (*panel b*). LDL-C (*panel a*) and TG (*panel c*) did not have a relationship with the probability of testing positive for SARS-CoV-2 infection [Pr (COVID test=1)]. The solid lines (—) represent the coefficient plot and the dashed lines (- -) represent the 95% confidence intervals. The p-values are calculated based on log-binomial regression.

The association of serum LDL-C, HDL-C, TC and TG levels with the risk of testing positive for SARS-CoV-2 infection using log-binomial regression is presented in Table 2. Compared to trajectory 1 of HDL-C, the trajectories 2 and 3 of antecedent HDL-C were associated with a significantly lower risk of SARS-CoV-2 infection in the unadjusted model (RR 0.85, 95%CI 0.76-0.96 and RR 0.56, 95%CI 0.41-0.76, respectively) and adjusted model 1 ((RR 0.86, 95%CI 0.76-0.96 and RR 0.57, 95%CI 0.42-0.78, respectively) (Table 2). Similarly, tertile 3 of HDL-C had a consistent significant lower risk of SARS-CoV-2 infection compared to tertile 1 in both the unadjusted (RR 0.77, 95%CI 0.67-0.87) and adjusted models (RR 0.76, 95%CI 0.67-0.88). Tertile 2 of HDL-C had a lower risk of infection compared to tertile 1 in one of the adjusted models (RR 0.86, 95%CI 0.77-0.98). Antecedent LDL-C and TC showed no significant association with the risk of testing positive for SARS-CoV-2 infection in both the trajectory and tertile models (Table 2). Tertile 2 of TG showed a higher risk of infection compared to tertile 1 of TG (RR 1.20, 95%CI 1.06-1.36), but this was not supported by the similar results in the corresponding trajectory models. The co-efficient plot for the association of antecedent lipid levels with the risk of SARS-CoV-2 infection is shown in Figure 3.

**Table 2:** Association of trajectories and tertiles of antecedent lipid levels with the risk of testing positive for COVID-19 using log-binomial regression.

Type of lipid	Trajectory	Unadjusted RR	Adjusted RRModel 1	Adjusted RRModel 2	Tertile	Unadjusted RR	Adjusted RR Model 1	Adjusted RR Model 2
LDL-C	Trajectory 1	Ref	Ref	Ref	Tertile 1	Ref	Ref	Ref
	Trajectory 2	1.06 [0.96-1.19]	1.05 [0.95-1.17]	1.05 [0.94-1.17]	Tertile 2	1.01 [0.89-1.15]	0.96 [0.84-1.09]	0.96 [0.84-1.10]
	Trajectory 3	1.17 [0.83-1.66]	1.08 [0.77-1.53]	1.07 [0.76-1.50]	Tertile 3	1.09 [0.96-1.24]	1.06 [0.93-1.20]	1.05 [0.93-1.20]
HDL-C	Trajectory 1	Ref	Ref	Ref	Tertile 1	Ref	Ref	Ref
	Trajectory 2	**0.85 [0.76-0.96]**	**0.86 [0.76-0.96]**	0.91 [0.80-1.02]	Tertile 2	0.89 [0.79-1.01]	**0.86 [0.77-0.98]**	0.91 [0.80-1.03]
	Trajectory 3	**0.56 [0.41-0.76]**	**0.57 [0.42-0.78]**	**0.64 [0.47-0.87]**	Tertile 3	**0.77 [0.67-0.87]**	**0.76 [0.67-0.88]**	**0.84 [0.73-0.96]**
TC	Trajectory 1	Ref	Ref	Ref	Tertile 1	Ref	Ref	Ref
	Trajectory 2	0.98 [0.88-1.09]	1.00 [0.90-1.12]	1.01 [0.90-1.12]	Tertile 2	0.96 (0.84-1.09)	0.96 (0.84-1.09)	0.95 (0.84-1.08)
	Trajectory 3	0.96 [0.71-1.30]	0.95 [0.71-1.28]	0.94 [0.70-1.27]	Tertile 3	0.96 (0.84-1.09)	0.98 (0.86-1.11)	0.98 (0.86-1.12)
TG	Trajectory 1	Ref	Ref	Ref	Tertile 1	Ref	Ref	Ref
	Trajectory 2	0.81 [0.65-1.01]	0.89 [0.73-1.10]	0.84 [0.68-1.03]	Tertile 2	1.13 [0.99-1.28]	**1.20 [1.06-1.36]**	**1.16 [1.02-1.32]**
	Trajectory 3	1.16 [0.79-1.69]	1.20 [0.83-1.74]	1.16 [0.80-1.67]	Tertile 3	0.99 [0.87-1.13]	1.13 [0.99-1.29]	1.04 [0.91-1.19]

Adjusted Model 1: Age + Gender + Race + CCI + Statin intensity + DHPCCB + nDHPCCB + ACEI + Alcohol intake + Times tested.

Adjusted Model 2: Model 1 + DM + HTN + HIV + BMI + Vaccination.

DHPCCB, dihydropyridine calcium channel blocker; nDHPCCB, non-dihydropyridine calcium channel blocker; CCI, Charlson comorbidity index; DM, diabetes mellitus; TC, Total Cholesterol; TG, Triglycerides; HIV, human immunodeficiency virus; HTN, hypertension; IQR, interquartile range; HDL-C, High density lipoprotein cholesterol; LDL-C, low density lipoprotein cholesterol; Ref, Reference group; RR, Relative risk of COVID-19 positivity.

#### Sensitivity and subgroup analysis

Sensitivity analysis for the risk of testing positive for SARS-CoV-2 infection using mixed-effects Poisson regression analysis, considering all the COVID-19 tests undertaken by each patient is presented in Supplementary Table 5A. Trajectory 3 (RR 0.62, 95%CI 0.48-0.86) and tertile 3 of HDL-C (RR 0.84, 95%CI 0.73-0.98) showed a consistently lower risk of infection compared to trajectory 1 and tertile 1 of HDL-C, respectively. Sensitivity analysis to assess the risk of testing positive for SARS-CoV-2 infection by considering antecedent lipid levels as continuous variables, showed that higher HDL-C levels had lower risk of COVID-19 positivity (RR 0.996, 95%CI 0.992-0.999), while LDL-C, TC and TG showed no relationship, in the adjusted analysis (Supplementary Table 5B). Sensitivity analysis of the association of the risk of testing positive for COVID-19 after adjusting for the setting of COVID-19 testing (asymptomatic screening or testing due to the presence of symptoms) is shown in Supplementary Table 5C. Trajectory 3 (RR 0.62, 95%CI 0.46-0.85) and tertile 3 (RR 0.84, 95%CI 0.73-0.96) of HDL-C had lower RR for COVID-19 positivity compared to Trajectory 1 and tertile 1 of HDL-C, respectively. Other lipid fractions did not show significant association with SARS-CoV-2 infection risk in this sensitivity analysis.

The subgroup analysis for the association between the different trajectories and tertiles of HDL-C and the risk of SARS-CoV-2 infection among the different trajectories and tertiles of LDL-C and TG are shown in Table 3. The risk of SARS-CoV-2 infection was consistently the lowest in the subgroups with the trajectory 3 or tertile 3 for HDL-C and trajectory 1 or tertile 1 for LDL-C (RR 0.53, 95%CI0.54-0.82 and RR 0.69, 95%CI 0.54-0.89), respectively. Similarly, the risk of SARS-CoV-2 infection was low in the subgroup with the trajectory 3 for HDL-C and trajectory 1 for TG (RR 0.55, 95%CI 0.40-0.75). Similar significant results were not obtained from the subgroup analysis using the tertile approach.

**Table 3:** Analysis of the association of trajectories and tertiles of antecedent HDL levels with the risk of testing positive for COVID-19 using log-binomial regression among the different subgroups of LDL-C and TG.

Type of lipid	Model	Trajectory	HDL-C			Tertile	HDL-C		
			Trajectory 1	Trajectory 2	Trajectory 3		Tertile 1	Tertile 2	Tertile 3
LDL-C	Unadjusted RR	Trajectory 1	Ref (1.0)	**0.79 (0.67-0.94)**	**0.53 (0.34-0.82)**	Tertile 1	Ref (1.0)	0.92 (0.75-1.14)	**0.69 (0.54-0.89)**
		Trajectory 2	1.04 (0.91-1.19)	0.83 (0.65-1.05)	**0.55 (0.34-0.88)**	Tertile 2	1.03 (0.84-1.27)	0.96 (0.67-1.36)	0.72 (0.49-1.04)
		Trajectory 3	1.09 (0.68-1.76)	0.87 (0.52-1.45)	0.57 (0.30-1.11)	Tertile 3	1.14 (0.93-1.40)	1.05 (0.74-1.49)	0.79 (0.55-1.14)
	Adjusted RR Model 1	Trajectory 1	Ref (1.0)	**0.80 (0.68-0.94)**	**0.53 (0.34-0.83)**	Tertile 1	Ref (1.0)	0.92 (0.75-1.14)	**0.69 (0.55-0.89)**
		Trajectory 2	1.02 (0.89-1.16)	0.81 (0.64-1.04)	**0.54 (0.34-0.87)**	Tertile 2	0.99 (0.81-1.21)	0.93 (0.65-1.29)	0.70 (0.48-1.00)
		Trajectory 3	0.99 (0.62-1.57)	0.79 (0.47-1.31)	0.55 (0.27-1.01)	Tertile 3	1.09 (0.89-1.34)	1.01 (0.72-1.43)	0.76 (0.53-1.11)
	Adjusted RR Model 2	Trajectory 1	Ref (1.0)	**0.85 (0.72-0.99)**	**0.59 (0.38-0.92)**	Tertile 1	Ref (1.0)	0.96 (0.78-1.18)	**0.76 (0.59-0.97)**
		Trajectory 2	1.01 (0.89-1.15)	0.86 (0.67-1.09)	**0.60 (0.37-0.96)**	Tertile 2	0.96 (0.78-1.18)	0.92 (0.65-1.29)	0.73 (0.50-1.06)
		Trajectory 3	0.98 (0.61-1.56)	0.83 (0.50-1.38)	0.58 (0.30-1.12)	Tertile 3	1.09 (0.89-1.33)	1.03 (0.74-1.47)	0.83 (0.57-1.19)
TG	Unadjusted RR	Trajectory 1	Ref	**0.84 (0.74-0.93)**	**0.55 (0.40-0.75)**	Tertile 1	Ref	0.92 (0.72-1.18)	**0.74 (0.59-0.95)**
		Trajectory 2	0.77 (0.62-1.01)	**0.65 (0.49-0.85)**	**0.42 (0.29-0.63)**	Tertile 2	1.11 (0.87-1.41)	1.02 (0.66-1.58)	0.83 (0.54-1.28)
		Trajectory 3	1.01 (0.68-1.51)	0.85 (0.55-1.31)	**0.56 (0.33-0.93)**	Tertile 3	0.94 (0.74-1.17)	0.86 (0.56-1.32)	0.70 (0.46-1.06)
	Adjusted RR Model 1	Trajectory 1	Ref (1.0)	**0.85 (0.76-0.96)**	**0.57 (0.42-0.78)**	Tertile 1	Ref (1.0)	0.93 (0.73-1.18)	0.80 (0.63-1.02)
		Trajectory 2	0.88 (0.71-1.09)	0.75 (0.58-1.00)	**0.50 (0.34-0.74)**	Tertile 2	1.21 (0.96-1.54)	1.13 (0.74-1.73)	0.98 (0.64-1.49)
		Trajectory 3	1.11 (0.75-1.64)	0.95 (0.62-1.44)	0.63 (0.38-1.05)	Tertile 3	1.11 (0.88-1.38)	1.03 (0.67-1.57)	0.89 (0.58-1.35)
	Adjusted RR Model 2	Trajectory 1	Ref	0.90 (0.79-1.02)	**0.64 (0.47-0.87)**	Tertile 1	Ref (1.0)	0.96 (0.75-1.22)	0.86 (0.68-1.09)
		Trajectory 2	0.84 (0.68-1.04)	0.76 (0.58-1.00)	**0.54 (0.36-0.80)**	Tertile 2	1.19 (0.94-1.51)	1.14 (0.74-1.74)	1.03 (0.67-1.57)
		Trajectory 3	1.09 (0.74-1.61)	0.99 (0.65-1.49)	0.70 (0.42-1.15)	Tertile 3	1.04 (0.83-1.30)	0.99 (0.65-1.51)	0.90 (0.59-1.36)

Adjusted Model 1: Age + Gender + Race + CCI + Statin intensity + DHPCCB + nDHPCCB + ACEI + Alcohol intake + Times tested.

Adjusted Model 2: Model 1 + DM + HTN + HIV + BMI + Vaccination.

DHPCCB, dihydropyridine calcium channel blocker; nDHPCCB, non-dihydropyridine calcium channel blocker; CCI, Charlson comorbidity index; DM, diabetes mellitus; TC, Total Cholesterol; TG, Triglycerides; HIV, human immunodeficiency virus; HTN, hypertension; IQR, interquartile range; HDL-C, High density lipoprotein cholesterol; LDL-C, low density lipoprotein cholesterol; Ref, Reference group; RR, Relative risk of COVID-19 positivity.

### Trends in serum lipid levels before, at the time of and after SARS-CoV-2 infection

**Figure 4:**
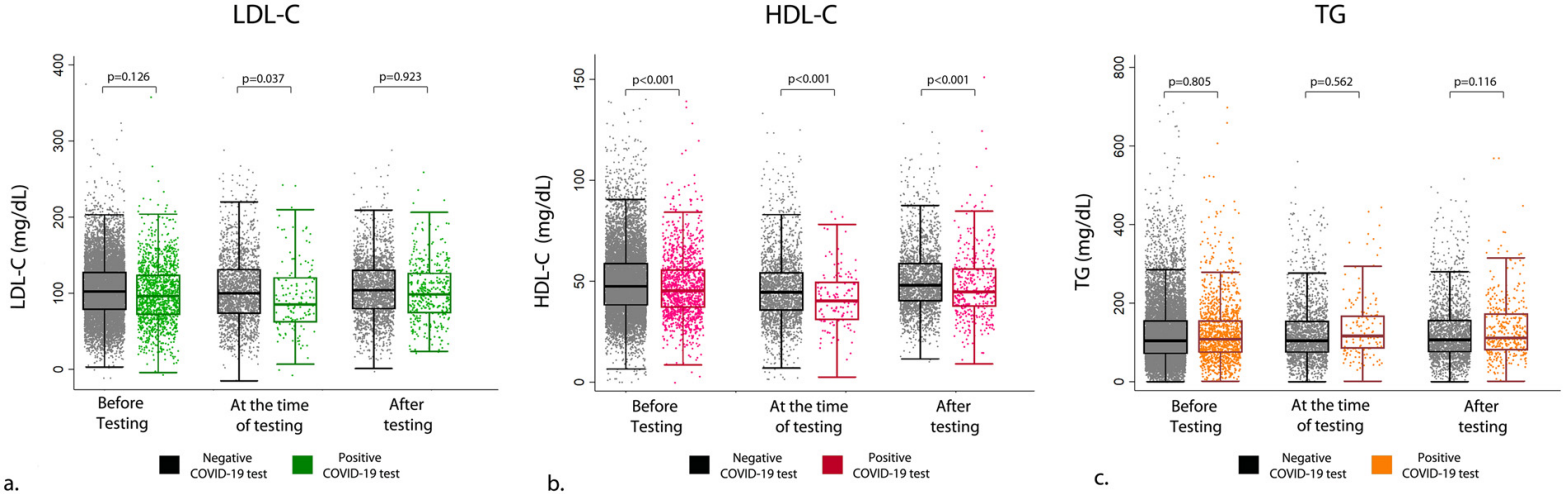
**Serum lipid levels before, at the time of, and after COVID-19 testing with trends assessed using mixed-effects linear regression analysis**. Antecedent to COVID-19 testing (N=9202) (730 days to 14 days prior to COVID-19 testing), HDL-C was significantly lower in the COVID-19 positive group, while antecedent LDL-C and TG were not different. At the time of testing (N=1886) (within 14 days before or after COVID-19 testing), LDL-C (*panel a*), and HDL-C levels (*panel b*) were significantly lower in COVID-19 positive patients while TG levels (*panel c*) were similar across the two groups. The median duration of follow-up subsequent to COVID testing is 261 (IQR 115 to 395) days. After 60 days follow-up post testing (N=2198), LDL-C, HDL-C and TG levels returned to nearly pre-infection levels among COVID-19 patients who survived.

Comparison of the trends in serum lipid levels before, at the time of and after COVID-19 testing (Figure 4) between COVID-19 positive and negative patients using mixed-effects linear regression analysis are shown in Table 4. The median duration of follow-up subsequent to COVID testing is 261 (IQR 115 to 395) days. LDL-C (−6.29, 95%CI −12.22 to −0.37 mg/dL), HDL-C (−7.7, 95%CI −9.8 to −5.5 mg/dL) and TC (−11.7, 95%CI −18.9 to −4.5 mg/dL) levels were significantly lower among COVID-19 infected individuals at the time of COVID-19 testing. LDL-C (−0.2, 95%CI −3.9 to 3.5 mg/dL), HDL-C (−1.9, 95%CI −3.2 to −0.6 mg/dL) and TC (−1.5, 95%CI −6.0 to 3.1 mg/dL) levels returned to pre-infection levels 6 months post-COVID-19 testing. In contrast, TG were not significantly different between COVID-19 positive and negative patients.

**Table 4:** Difference in lipid levels between COVID-19 positive and negative individuals before, at the time of and 60-days after testing for SARS-CoV-2 infection using mixed-effects linear regression analysis.

Type of Lipid	Time of Lipid testing	Unadjusted		Adjusted Model 1		Adjusted Model 2	
		β-coefficient [95%CI] mg/dL	p-value	β-coefficient [95%CI] mg/dL	p-value	β-coefficient [95%CI] mg/dL	p-value
LDL-C	Before Testing	1.8 [−0.51 to 4.12]	0.126	0.70 [−1.58 to 2.98]	0.434	0.86 [−1.41 to 3.14]	0.458
	At the time of Testing	−6.29 [−12.22 to -0.37]	**0.037**	−6.73 [−12.58 to -0.89]	**0.030**	−6.45 [−12.3 to -0.58]	**0.031**
	After Testing	−0.18 [−3.90 to 3.54]	0.923	−0.58 [−4.28 to 3.11]	0.876	−0.39 [−4.07 to 3.30]	0.837
HDL-C	Before Testing	−2.02 [−2.99 to -1.03]	**<0.001**	−1.84 [−2.77 to -0.92]	**<0.001**	−1.19 [−2.10 to -0.28]	**0.010**
	At the time of Testing	−7.7 [−9.8 to -5.49]	**<0.001**	−7.21 [−9.31 to -5.12]	**<0.001**	−6.58 [−8.66 to -4.49]	**<.0.001**
	After Testing	−1.9 [−3.2 to -0.56]	**0.005**	−1.63 [−2.93 to -0.34]	**0.020**	−0.99 [−2.28 to 0.29]	0.129
TC	Before Testing	−0.71 [−3.5 to 2.12]	0.622	−0.8 [−3.6 to 1.9]	0.573	−0.6 [−3.4 to 2.1]	0.652
	At the time of Testing	−11.71 [−18.9 to -4.5]	**0.001**	−11.4 [−18.5 to -4.3]	**0.002**	−11.0 [−18.1 to 3.9]	**0.002**
	After Testing	−1.5 [−6.0 to 3.1]	0.531	−0.6 [−5.1 to 3.9]	0.806	−0.4 [−4.9 to 4.1]	0.869
TG	Before Testing	−0.93 [−8.31 to 6.45]	0.805	1.95 [−5.33 to 9.23]	0.600	−0.50 [−7.75 to 6.76]	0.893
	At the time of Testing	5.99 [−14.27 to 26.26]	0.562	3.98 [−16.01 to 23.97]	0.696	2.60 [−17.38 to 22.59]	0.799
	After Testing	10.29 [−2.54 to 23.12]	0.116	12.25 [−0.44 to 24.95]	0.058	10.40 [−2.26 to 23.07]	0.107

Adjusted Model 1: Age + Gender + Race + CCI + Statin intensity + DHPCCB + nDHPCCB + ACEI + Alcohol intake.

Adjusted Model 2: Model 1 + DM + HTN + HIV + BMI.

B, linear regression co-efficient; 95%CI – 95% Confidence interval.

DHPCCB, dihydropyridine calcium channel blocker; nDHPCCB, non-dihydropyridine calcium channel blocker; CCI, Charlson comorbidity index; DM, diabetes mellitus; TC, Total Cholesterol; TG, Triglycerides; HIV, human immunodeficiency virus; HTN, hypertension; IQR, interquartile range; HDL-C, High density lipoprotein cholesterol; LDL-C, low density lipoprotein cholesterol; Ref, Reference group; RR, Relative risk of COVID-19 positivity.

### COVID-19 outcomes

There was no significant association of antecedent lipid levels with admission for SARS-CoV-2 infection by log-binomial regression (Supplementary table 6). Antecedent lipid levels were not associated with the development of severe COVID-19 disease, and mortality following SARS-CoV-2 infection by log-binomial regression and cox-proportional hazards regression, respectively (Supplementary Tables 7 and 8).

### Levels of inflammatory markers following COVID-19 diagnosis

Tertile 3 of HDL-C was associated with lower levels of CRP (-34.4 ± 12.5 mg/dL) using multilevel mixed effect linear regression analysis (Supplementary Table 9). Similar associations were not observed in the analysis based on HDL-C trajectories (-2.9 ± 45.2 mg/dL). Antecedent LDL-C, TC and TG levels were not associated with post-COVID-19 diagnosis CRP levels. Similarly, antecedent lipid levels were not associated with the levels of other inflammatory markers, such as ESR, procalcitonin, and IL-6 levels after COVID-19 diagnosis (Supplementary Table 10).

## Discussion

**Figure 5:**
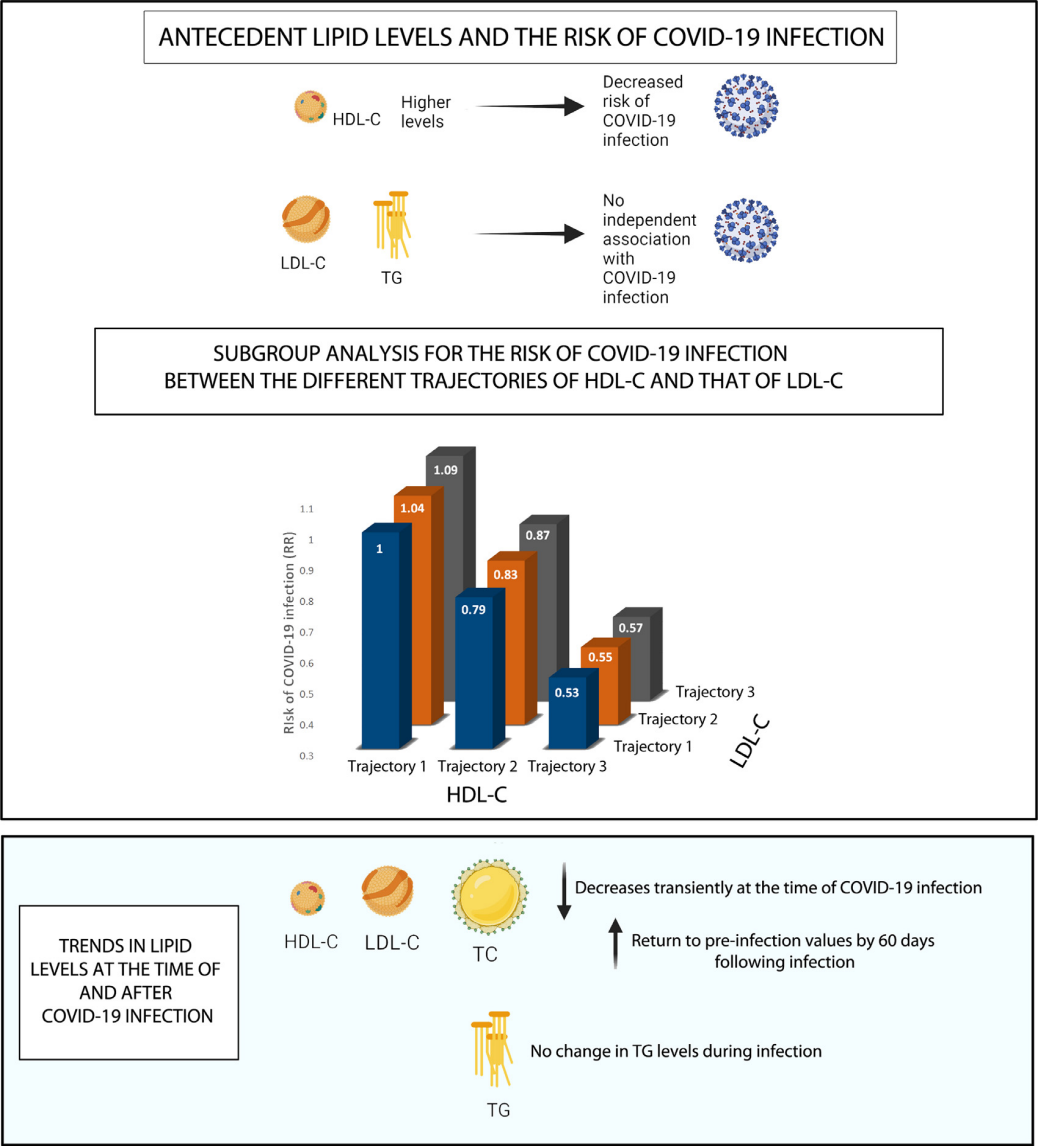
**Summary of Results**. The *top panel* - association of antecedent lipid levels with the risk of COVID-19. The *middle panel* - subgroup analysis of the risk of SARS-CoV-2 infection for the 3 trajectories of HDL-C among the 3 trajectories of LDL-C. The *bottom panel* shows the trends in lipid levels during and after COVID-19.

In our study, patients in the higher trajectories and tertiles of HDL-C had lower risks of SARS-CoV-2 infection independent of other confounders. These results were consistent in the sensitivity analyses using mixed-effects Poisson regression, including all the available COVID-19 tests for each patient. Antecedent levels of LDL-C, TC, and TG were not independently associated with the risk of SARS-CoV-2 infection. However, during subgroup analysis, the risk of COVID-19 was consistently the lowest in the subgroup with the highest HDL-C and the lowest LDL-C levels in both the trajectory- and tertile-based models. Additionally, HDL-C, LDL-C, and TC levels declined transiently at the time of COVID-19 diagnosis and later returned to pre-infection levels by two months after SARS-CoV-2 infection (Figure 5). Among COVID-19 positive patients, the highest HDL-C tertile had lower CRP levels following COVID-19 diagnosis. Among COVID-19 patients, there was no significant association between any of the antecedent lipid levels and the risk of hospitalization due to COVID-19, disease severity, mortality, or other inflammatory parameters (such as IL-6, ESR and procalcitonin), which may be due to inadequate power to assess these associations.

Comorbidities like HTN, DM, and obesity have been identified as important prognostic factors among patients admitted with COVID-19.^3^^,^^23^ In a recent meta-analysis, low serum LDL-C, HDL-C, and TC, but not TG, levels during hospital admission were associated with the development of severe SARS-CoV-2 infection and mortality.^7^ Two studies using UK biobank data^16^^,^^24^ evaluated the association of antecedent serum lipid levels with the development and prognosis of COVID-19. But the serum lipid levels were measured ten years prior to COVID-19 testing and may not accurately reflect current values. Of these studies, while the case definition was restricted to hospitalized COVID-19 patients with severe symptoms in the first study^16^; the context for COVID-19 testing and the disease severity of the included patients were unclear in the second one.^24^ Despite these limitations, these studies^16^^,^^24^ found an inverse association between serum HDL-C and risk of SARS-CoV-2 infection, similar to our findings. Mendelian randomization (MR) studies evaluating the association of genetically determined lipid levels, and SARS-CoV-2 infection have yielded conflicting results,^16^^,^^25^ with one study showing a lack of association^16^ and the other study demonstrating a greater risk of infection with higher LDL-C and TC levels.^25^ Although MR is a valuable tool to assess causal relationships, it is not without its limitations. The genetic instruments in MR studies are surrogate measures of lifelong changes in circulating lipids and might not indicate short-term changes secondary to pharmacological interventions. Furthermore, almost all previously recognized genetic variants for HDL-C levels have some degree of pleiotropic relationships with other lipid or metabolic traits.^26^ Such horizontal pleiotropy may either lead to false-positive causal associations or decrease the power to detect existing associations.^27^

Despite contrasting reports, substantial evidence supports the biological plausibility of our findings. Low HDL-C levels have been associated with an increased risk of infections, hospitalization, and infection-related and all-cause mortality in multiple epidemiological^13^^,^^28^^,^^29^ and genetic studies.^28^ Though a U-shaped relationship between HDL-C and the risk of infections in general was described in a study from two population cohorts, the risk was still much lower in patients with elevated HDL-C compared to patients with HDL-C lower than 45 mg/dL. HDL was shown to have important immunomodulatory properties beyond cholesterol reverse transportation^11^ and could be altered in COVID-19.^30^ The anti-inflammatory effects of HDL could be due to both cholesterol efflux-dependent^31^ and independent mechanisms.^32^ A CETP (cholesteryl ester transfer protein) gain-of-function variant was associated with significant reductions in HDL-C levels during sepsis, and increased risk of mortality.^33^ Conversely, patients with a genetic score indicating decreased CETP function had significantly reduced sepsis-related mortality in the UK Biobank and iSPAAR cohorts, and mouse models of sepsis treated with the CETP inhibitor anacetrapib had higher HDL-C levels and better survival relative to those treated with placebo.^34^

Preclinical studies have highlighted the importance of HDL in viral infections,^35^ specifically SARS-CoV-2 infection, and glycation of HDL has been shown to impair its antiviral activity. Lipid rafts, which are cholesterol-rich microdomains on host cell membranes, play a vital role in viral entry and budding.^36^ LDL promotes lipid raft formation,^37^ and it is possible that HDL depletes cholesterol in lipid rafts through cholesterol efflux from cells.^38^ In a preclinical study, depletion of cell-membrane cholesterol decreased the risk of SARS-CoV2 infection by decreasing the trafficking of ACE2 and furin protease to the lipid rafts.^39^ Additionally, scavenger receptor protein-B1 (SR-B1), which is an HDL receptor, has been shown to facilitate the ACE2-dependent entry of SARS-CoV-2.^40^ *In vitro* studies indicate that lower concentrations of HDL-C promote SRB1-mediated SAR-CoV-2 infection, while higher HDL-C concentrations inhibit SARS-CoV-2 infection.^41^ Apo-A1, an important component of HDL, is shown to inhibit viral fusion and entry into host cells.^42^ Taken together, these data support our findings that increased serum HDL-C may to be protective against SARS-CoV-2 infection.

Our study has several strengths. We were able to obtain data on recent lipid levels prior to COVID-19 testing, and the robustness of our results was assessed by using both the group-based trajectory modelling and tertiles of weighted mean approach. Our subgroup analysis of the association of HDL-C across the different trajectories and tertiles of LDL-C and TG showed the importance of the concomitant effect of these lipid fractions. Importantly, we demonstrated that the decline in serum LDL-C and TC is transient and likely secondary to SARS-CoV-2 infection, and that low LDL-C and TC are not predisposing factors for the development of infection. Our results were consistent even after adjusting for the reason for COVID-19 testing (symptomatic testing or asymptomatic screening), as shown in the sensitivity analysis. Our study population had near equal proportions of White and African American population, thus our results are more generalizable. Our analysis was adjusted for the use of lipid-lowering agents, which was not performed in the previous studies analysing this association. Our finding that higher HDL-C levels antecedent to SARS-CoV-2 infection were associated with lower levels of CRP affirms the importance of HDL-C levels in modulating inflammation.

Our study is also limited by several factors. We did not have access to serum lipid levels consistently in all the individuals in the two years prior to COVID-19 testing and we did not have adequate data on the lipid levels before and after initiation of lipid-lowering agents, such as statins. We lacked serum lipid measurements for all individuals at the time of COVID-19 testing and after 60 days post-testing. Lipid measurements were not confined to a fasting state, and thus diet may have influenced these values. Our study might also be limited by selection bias as we may not have sufficient data on younger individuals with a lower cardiovascular risk profile, because they may not have had serum lipid levels assessed routinely. We did not have information on lp(a), and apolipoproteins such Apo B and Apo A1 in our database. Additionally, we were unable to assess certain properties of HDL in our study, such as Serum amyloid-A enrichment or paraoxonase-1 activity, which have been shown to be associated with COVID-19 even in the early stages of infection. We did not have sufficient power to detect the association of antecedent lipid levels with the development of severe disease and COVID-19 related mortality. Furthermore, our inferences might be susceptible to bias from residual confounding due to the retrospective observational study design.

Our findings have important clinical applications due to a potential causal relationship between low HDL-C levels and susceptibility to SARS-CoV-2 infection. The serum lipid levels were measured at least two weeks prior to COVID-19 testing, and this temporal association enabled our model to be less susceptible to reverse-causality. Prior studies have consistently shown a similar association of greater disease severity in infectious diseases with lower HDL-C levels, which is analogous to our current scenario. We also demonstrate a biological gradient as the HDL-C levels become lower. These findings raise the intriguing possibility that drugs that raise HDL-C levels, such as CETP inhibitors, could be beneficial in the prevention and treatment of COVID-19, and perhaps infectious diseases in general.^31^ Though some studies on HDL-C-raising agents, such as torcetrapib, reported a non-significant increase in the risk of infectious diseases in the CETP inhibitor arm,^43^ this was not observed in other trials with CETP inhibitors.^44^^,^^45^ Likewise, treatment with statins, especially rosuvastatin, has been shown to have a dose-dependent increase in the HDL-C levels in addition to its LDL-C-lowering effects. A recent meta-analysis of cohort studies has shown that statin use reduces mortality in SARS-CoV-2 infection.^46^ Additional experimental evidence is needed to determine whether increasing serum levels of HDL-C can have a salutary effect vis-a-vis decreasing the susceptibility to SARS-CoV-2 infection or improving COVID-19-related outcomes.^15^

In conclusion, higher antecedent HDL-C levels, especially in the subgroup with low LDL-C and TG, decrease the SARS-CoV-2 infection risk. We believe that this relationship may be causal. LDL-C, TG, and TC were not independently associated with SARS-CoV-2 infection. A decline in serum HDL-C, LDL-C, and TC at the time of infection is transient, with a return to pre-infection levels by 60 days post-infection. Additionally, low HDL-C levels were associated with low CRP levels during the course of the illness; thus, strengthening the role of HDL-C in the regulation of inflammation. The results of our study could provide the impetus for clinical trials of interventions aimed at increasing HDL-C levels in the prevention and amelioration of SARS-CoV-2 infection or infections in general.

## Contributors

VC and JLM conceived the idea for the study. VC, AK, MM, and RS designed and undertook the literature review. AB, AKi and KG performed the data curation for the study. VC performed the statistical analysis. VC, AKi and KG have accessed and verified the underlying data VC, AK created the figures. VC, AK, MM, BS, RK, DV, MB, PG, SA, PK and JLM wrote the first draft of the manuscript. VC, AK, MM, BS, AB, AKi, KG, PG, SA, PK and JLM revised the subsequent drafts of the manuscript. All authors reviewed the final draft of the manuscript.

## Data sharing statement

Primary de-identified data will be provided at reasonable request made to the corresponding author.

## Declaration of interests

We declare no conflicts of interest.

## Appendix Supplementary materials

Image, application 1

Image, application 2
