# Supplementary material for: HDL cholesterol levels and susceptibility to COVID-19
Source: eBioMedicine. 2022 Jul 15;82:104166. doi: 10.1016/j.ebiom.2022.104166 (PMC9284176; doi:10.1016/j.ebiom.2022.104166)
Supplement: Supplementary file 1 [file mmc1.pdf]

SUPPLEMENTARY APPENDIX

SUPPLEMENTARY METHODS

Comorbidities

Tobacco use was defined as at least five cigarettes per week, almost every week for at least three months. Hypertension was defined as systolic blood pressure (BP) ≥130 mm Hg, diastolic BP≥80 mm Hg, and/or the use of antihypertensive medication(s). DM was defined as fasting glucose ≥ 126 mg/dl or hemoglobinA1C (HbA1C) > 6.5 and/or use of insulin or oral hypoglycemic medication(s). Obesity was defined as a BMI ≥ 30 kg/m^2.

Admission and In-hospital outcomes

We assessed the length of hospital stay, and the level of oxygen therapy, and other supportive measures required. The length of hospital stay was defined as the duration between the date of the index admission and the date of final hospital discharge, in-hospital death, or administrative censorship on December 15, 2021. We characterized the requirement for oxygen therapy as no requirement, requirement of oxygen support using nasal cannula, high-flow oxygen, noninvasive positive pressure ventilation (NIPPV), or mechanical ventilation. We noted the highest oxygen support required in the order described above. Other supportive measures recorded were extracorporeal membrane oxygenation (ECMO), vasopressor agents, and renal replacement therapy.

Statistical analysis

Multivariable adjustment was made for age, gender, race, BMI, smoking, alcohol use, DM, HTN, lipid-lowering therapy, estimated glomerular filtration rate (eGFR), Charlson comorbidity index, intensity of statin therapy(24), dihydropyridine calcium channel blocker (DHP-CCB), non-dihydropyridine calcium channel blocker (nDHP-CCB), angiotensin converting enzyme inhibitors (ACEI) and the number of times the individual was tested for COVID-19.

We also analyzed the association of antecedent serum lipid levels with the longitudinal data of available inflammatory markers, namely CRP, ESR, IL-6, and procalcitonin, in the first 60 days after COVID-19 diagnosis using mixed-effects linear regression analyses. We used multiple imputations with chained equations to fill out the missing lab values.

SUPPLEMENTARY RESULTS

Comorbidities

The common comorbidities in our cohort were HTN (75.8%), DM (35.8%), HIV infection (1.5%), congestive heart failure (17.6%), coronary artery disease (28.7%), CKD > stage 2 (21.25%), obesity (BMI>30) (48.9%), hypothyroidism (17.3%), and solid organ or hematological malignancy (13.2%). Forty-three percent of this population were ever-smokers, and 2.7% reported alcohol use disorder.

Baseline characteristics stratified by lipid tertiles and trajectories

Patient characteristics stratified by trajectories of antecedent serum lipid levels are shown in **Supplementary Tables 1A and 1B**. Patients in the LDL-C trajectory 1 had a higher proportion of males compared to trajectories 2 and 3 (43.5% vs 33.9% vs 24.9%, p<0.001). Obesity (BMI>30) was less prevalent in the LDL-C trajectory-1 compared to the trajectories 2 and 3 (median 48.1% vs 51.5% vs 50.8%, p=0.007). The comorbidities were higher in LDL-C trajectories 1 and 3 compared to trajectory 2 for congestive heart failure (20.7% vs 12.8% vs 17.5%, p<0.001), DM (42.1% vs 29.6%, 41.3%, p<0.001), HTN (79.1% vs 71.7% vs 74.6%, p<0.001), CKD Stage 3–5 (26.6% vs. 17.1% vs. 24.3%, p-value = 0.004) and alcohol use disorder (10.5% vs 7.7% vs 10.1%, p<0.001).

Patients in the HDL-C trajectory 1 had a higher proportion of males compared to trajectories 2 and 3 (48.8% vs 24.5% vs 20.8%, p<0.001). The comorbidities were highest in the HDL-C trajectory 1 compared to trajectories 2 and 3 for obesity (BMI>30) (56.0% vs 42.1% vs 21.8%, p<0.001), congestive heart failure (19.4% vs 14.3% vs 12.9%, p<0.001), DM (42.9% vs 28.6% vs 23.4%, p<0.001), HTN (79.1% vs 70.7% vs 72.3%, p<0.001), and CKD Stage 3–5 (24.9% vs. 19.5% vs. 17.6%, p-value = 0.004).

Alcohol use disorder was highest in the trajectory 3 of HDL-C (9.1% vs 8.2% vs 19.2%, p<0.001) **(Supplementary Table 1A)**. Baseline characteristics stratified by TC and TG trajectories are available in **Supplementary Table 1B.**

In-hospital outcomes

Among hospitalized patients, 115 patients (31.4%) did not require any form of supplemental oxygen (O_2), 143 patient (39.2%) required O_2 via nasal cannula, 44 patients (12.1%) required O_2 via high flow oxygen, 13 patients (3.6%) required O_2 via noninvasive ventilation as the highest level of oxygen support, while 51 (13.9%) patients required intubation and mechanical ventilation. 115 patients (31.4%) did not require any form of oxygen requirement throughout their hospitalization. There were no significant differences in O_2 requirement and other supportive therapy required for patients across the different antecedent LDL-C, HDL-C, TC and TG trajectories or tertiles **(Table 2A and 2B).**

SUPPLEMENTARY TABLES

Supplementary table 1A: Baseline Characteristics of patients based on the trajectories of LDL-C and HDL-C during the 2 years antecedent to COVID-19 testing.

Characteristics	Unit	Total (N=11001)	LDL-C Trajectory 1 (N=5157)	LDL-C Trajectory 2 (N=3709)	LDL-C Trajectory 3 (N=189)	p-value	HDL-C Trajectory 1 (N=5687)	HDL-C Trajectory 2 (N=2971)	HDL-C Trajectory 3 (N=495)	p-value
Age	Median (IQR)	59 (46-70)	61 (47-71)	57 (45-67)	59 (49-66)	0.676	58(45-68)	60(47-71)	61(42-71)	0.008
Male Sex (%)	n (%)	4486 (40.8%)	2250 (43.5%)	1259 (33.9%)	47 (24.9%)	<0.001	2275(48.8%)	728(24.5%)	103(20.8%)	<0.001
Race	n (%)					0.056				0.014
White		5875 (53.4%)	2697 (52.1%)	1913 (51.5%)	83 (43.9%)		2978 (52.4%)	1532 (51.6%)	248 (50.1%)	
AA		4410 (40.1%)	2173(41.9%)	1548 (41.7%)	100 (52.9%)		2328 (40.9%)	1272 (42.8%)	228 (46.1%)	
Hispanic		381 (3.46%)	164 (3.2%)	121 (3.3%)	2 (1.1%)		205 (3.6%)	75 (2.5%)	12 (2.4%)	
Native American		99 (0.9%)	43 (0.8%)	35 (0.9%)	2(1.1%)		58 (1.0%)	21(0.7%)	1(0.2%)	
Asian		202(1.8%)	93 (1.8%)	85 (2.3%)	1(0.5%)		105 (1.9%)	67 (2.3%)	6(1.2%)	
Unknown		34 (0.3%)	8 (0.2%)	10 (0.3%)	1(0.5%)		14 (0.3%)	5(0.2%)	0 (0%)	
BMI	Median (IQR)	29.7 (25.2-35.4)	29.6 (25.1-35.4)	30.3 (25.7-35.9)	30.1 (25.7-34.1)	0.001	31 (26.6-36.6)	28.3 (24.1-34.1)	25.1 (21.9-29)	<0.001
Obesity % (BMI ≥ 30)	n (%)	5341 (48.9%)	2486 (48.1%)	1909 (51.5%)	96 (50.8%)	0.007	3181 (56.0%)	1250 (42.1%)	108 (21.8%)	<0.001
Comorbidities										
HIV	n (%)	165(1.5%)	94 (1.8%)	52 (1.4%)	1 (0.5%)	0.152	102 (1.9%)	41 (1.4%)	5 (1.0%)	0.191
Alcohol	n (%)	1010 (9.2%)	546 (10.5%)	284 (7.7%)	19 (10.1%)	<0.001	517 (9.1%)	243 (8.2%)	95 (19.2%)	<0.001
Heart failure	n (%)	1939 (17.6%)	1070 (20.7%)	474 (12.8%)	33 (17.5%)	<0.001	1106 (19.4%)	424 (14.3%)	64 (12.9%)	<0.001
Chronic lung disease	n (%)	2260 (20.5%)	1180 (22.8%)	711 (19.2%)	38 (20.1%)	<0.001	1209 (21.3%)	629 (21.2%)	103 (20.8%)	0.971
DM	n (%)	3934 (35.8%)	2182 (42.1%)	1097 (29.6%)	78 (41.3%)	<0.001	2438 (42.9%)	850 (28.6%)	116 (23.4%)	<0.001
HTN	n (%)	8342 (75.8%)	4094 (79.1%)	2662 (71.7%)	141 (74.6%)	<0.001	4498 (79.1%)	2101 (70.7%)	358 (72.3%)	<0.001
Hypothyroidism	n (%)	1908 (17.3%)	937 (18.1%)	681 (18.4%)	38 (20.1%)	0.763	961 (16.9%)	599 (20.2%)	110 (22.2%)	<0.001
Liver disease	n (%)	1717 (15.6%)	949 (18.3%)	537 (14.5%)	40 (21.2%)	<0.001	1085 (19.1%)	400 (13.5%)	64 (12.9%)	<0.001
PVD	n (%)	1527 (13.8%)	899 (17.4%)	384 (10.3%)	26 (13.8%)	<0.001	900 (15.8%)	357 (12.0%)	65 (13.1%)	<0.001
CKD	n (%)	2338 (21.25%)	1375 (26.6%)	633 (17.1%)	46 (24.3%)	<0.001	1414 (24.9%)	579 (19.5%)	87 (17.6%)	<0.001
Malignancy	n (%)	1514 (13.17%)	784 (15.1%)	504 (13.6%)	26 (13.8%)	0.114	817 (14.4%)	434 (14.6%)	71 (14.3%)	0.954
CAD	n (%)	3156 (28.7%)	1743 (33.7%)	769 (20.7%)	57 (30.2%)	<0.001	1765 (31.0%)	727(24.5%)	101 (20.4%)	<0.001
AMI	n (%)	991 (9.0%)	512 (9.9%)	209 (5.6%)	16 (8.5%)	<0.001	541 (9.5%)	176 (5.9%)	29 (5.9%)	<0.001
CCI (0-24)	Median (IQR)	5 (3-8)	6 (3-9)	4(2-7)	6 (3-8)	<0.001	6 (3-8)	5 (2-8)	5 (2-7)	<0.001
Van Elixhauser index	Median (IQR)	5 (0-14)	6(0-16)	2(-2 – 11)	6(0-14)	<0.001	5(0-15)	3(0-12)	4(0-14)	<0.001
Statin use	n (%)	2823 (25.7%)	1374 (26.5%)	591 (15.9%)	54(28.6%)	<0.001	1458 (25.6%)	506(17.0%)	80 (16.2%)	<0.001
Low		93(0.9%)	48 (0.9%)	26 (0.7%)	2 (1.1%)	<0.001	42 (0.7%)	30 (1.0%)	4 (0.8%)	<0.001
Intermediate		723 (6.6%)	458 (8.9%)	153 (4.1%)	11 (5.8%)		424 (7.5%)	170 (5.7%)	33 (6.7%)	
High		2007(18.2%)	868 (16.8%)	412 (11.1%)	41 (21.7%)		992 (17.4%)	306 (10.3%)	43 (8.7%)	
DHP CCB	n (%)	2023 (18.4%)	971 (18.8%)	531 (14.3%)	43 (22.8%)	<0.001	1066 (18.7%)	413 (13.9%)	81 (16.4%)	<0.001
Non DHP CCB	n (%)	628 (5.7%)	325 (6.3%)	165 (4.5%)	5 (2.7%)	<0.001	347 (6.1%)	131 (4.4%)	21 (4.2%)	0.002
ACE inhibitors	n (%)	1156 (10.5%)	527 (10.2%)	293 (7.9%)	12 (6.4%)	<0.001	620 (10.9%)	180 (6.1%)	38 (7.7%)	<0.001
Labs										
Albumin (g/dL)	Median (IQR)	3.8(3.5-4.0)	3.7(3.4-4.0)	3.9(3.6-4.1)	3.8(3.4-4.0)	<0.001	3.8(3.4-4.0)	3.9(3.6-4.1)	3.9(3.7-4.1)	<0.001
AST (units/L)	Median (IQR)	22.6(18.7-28.7)	23(19-29)	22(18.5-27.5)	21.3(18-18.8)	0.060	22.5(18.6-29)	22(19-27)	24.3(20-32)	0.070
ALT (units/L)	Median (IQR)	21(16-29)	21(16.2-29)	21(16-29)	19.5(14.8-28.0)	0.262	22(16.6-31)	20(16-26)	20.5(16-27.8)	<0.001
INR	Mean (SD)	1.21(0.44)	1.23(0.46)	1.18(0.43)	1.19(0.38)	0.011	1.1(1-1.3)	1.1(1-1.1)	1.0(1-1.1)	<0.001
Creatinine (mg/dL)	Median (IQR)	0.95(0.8-1.2)	1(0.-8-1.28)	0.9(0.75-1.1)	0.98(0.8-1.27)	<0.001	1(0.8-1.3)	0.9(0.8-1.1)	0.9(0.7-1.1)	<0.001
BUN (mg/dL)	Median (IQR)	17.3(10.4-19)	14.5(11-20.6)	13(10-17)	14.1(9.5-19)	<0.001	14.3(10.7-20)	13.3(10-18)	13(10-16.7)	<0.001
eGFR (mL/min/1.73m^2)	Median (IQR)	73 (49.1-89.7)	72.1(44.6-89.3)	73.9 (55.3-89.9)	69.4(54.5-90)	0.127	72.8(48-5-89.9)	73.4(50.3-89.8)	74.6(60.2-83.8)	0.447

AA, African American; ALT Alanine transaminase; AMI, History of Acute myocardial infarction, AST, Aspartate transaminase; BMI, body mass index; BUN – Blood Urea Nitrogen; CAD, coronary artery disease; CAD, Coronary artery disease; CCI, Charlson comorbidity index; CKD, chronic kidney disease (≥Stage 3); DHP CCB, dihydropyridine calcium channel blocker; non DHP CCB, non-dihydropyridine calcium channel blocker; DM, diabetes mellitus; eGFR, Estimated Glomerular filtration rate; INR – International normalized ratio, IQR, Interquartile range; HDL-C, high density lipoprotein cholesterol; HIV, human immunodeficiency virus; HTN, hypertension; IQR, interquartile range; LDL-C, low density lipoprotein cholesterol; PVD, peripheral vascular disease; SD, standard deviation.

Supplementary table 1B: Baseline Characteristics of patients based on trajectories of TC and TG levels during the 2 years antecedent to COVID-19 testing.

Characteristics	Unit	Total (N=11001)	TC			p-value	TG			p-value
			Trajectory 1 (N=4370)	Trajectory 2 (N=4494)	Trajectory 3 (N=507)		Trajectory 1 (N=8187)	Trajectory 2 (N=828)	Trajectory 3 (N=150)	
Age	Median (IQR)	59 (46-70)	60(46-71)	58(46-68)	57(48-66)	<0.001	59(46-70)	58(46-68)	52.5(43-61)	0.021
Male Sex (%)	n (%)	4486 (40.8%)	2050(46.9%)	1490(33.1%)	73(23.8%)	<0.001	3123(38.1%)	399(48.2%)	90(60%)	<0.001
Race	n (%)					0.006				<0.001
White		5875 (53.4%)	2194(50.2%)	2408(53.6%)	165(53.8%)		4093(49.9%)	564(68.1%)	104(69.3%)	
AA		4410 (40.1%)	1924 (44.0%)	1787(39.8%)	125(40.7%)		3621(44.2%)	183(22.1%)	31(20.7%)	
Hispanic		381 (3.46%)	138(3.2%)	146 (3.3%)	8(2.6%)		237(2.9%)	44(5.3%)	11(7.3%)	
Native American		99 (0.9%)	38(0.9%)	38(0.9%)	4(1.3%)		65(0.8%)	13(1.6%)	2(1.3%)	
Asian		202(1.8%)	69(1.6%)	107(2.4%)	4(1.3%)		154(1.9%)	23(2.8%)	2(1.3%)	
Unknown		34 (0.3%)	7(0.2%)	10(0.2%)	1(0.3%)		18(0.2%)	1(0.1%)	0(0%)	
BMI	Median (IQR)	29.7 (25.2-35.4)	30(25.6-35.6)	29.8(25.2-35.6)	29.7(25.4-34.2)	0.164	29.7(25.2-35.5)	31(27.3-36.3)	31.1(27.9-36.2)	<0.001
Obesity % (BMI ≥ 30)	n (%)	5341 (48.9%)	2189(50.2%)	2209(49.2%)	150(48.9%)	0.624	3983(48.7%)	472(57.1%)	90(60.4%)	<0.001
Comorbidities										
HIV	n (%)	165(1.5%)	76(1.7%)	70(1.6%)	2(0.7%)	0.314	120(1.6%)	16(1.9%)	2(1.3%)	0.727
Alcohol	n (%)	1010 (9.2%)	437(10%)	386(8.6%)	31(10.1%)	0.064	754(9.2%)	84(10.1%)	18(12%)	0.358
Heart failure	n (%)	1939 (17.6%)	955(21.9%)	589(13.1%)	50(16.3%)	<0.001	1425(17.4%)	131(15.8%)	35(23.3%)	0.078
Chronic lung disease	n (%)	2260 (20.5%)	982 (22.5%)	898 (19.9%)	66 (21.5%)	0.016	1719 (20.9%)	189 (22.8%)	63 (24.0%)	0.329
DM	n (%)	3934 (35.8%)	1909(46.7%)	1371(30.5%)	128(41.7%)	<0.001	2892(35.3%)	427(51.6%)	85(56.7%)	<0.001
HTN	n (%)	8342 (75.8%)	3464(79.3%)	3267(72.7%)	237(77.2%)	<0.001	6136(74.9%)	707(85.4%)	119(79.3%)	<0.001
Hypothyroidism	n (%)	1908 (17.3%)	752(17.2%)	838(18.6%)	81(26.4%)	<0.001	1468(17.9%)	174(21.0%)	28(18.7%)	0.090
Liver disease	n (%)	1717 (15.6%)	800(18.3%)	687(15.3%)	60(19.5%)	<0.001	1322(16.2%)	187(22.6%)	27(24.7%)	<0.001
PVD	n (%)	1527 (13.8%)	778(17.8%)	509(11.3%)	35(11.4%)	<0.001	1169(14.3%)	125(15.1%)	26(17.3%)	0.479
CKD	n (%)	2338 (21.25%)	1185(27.1%)	817(18.2%)	78(25.4%)	<0.001	1780(21.7%)	246(29.7%)	51(34%)	<0.001
Malignancy	n (%)	1514 (13.17%)	660(15.1%)	622(13.8%)	43(14.0%)	0.231	1177(14.4%)	127(15.3%)	16(10.7%)	0.318
CAD	n (%)	3156 (28.7%)	1540 (35.2%)	975 (21.7%)	81 (26.4%)	<0.001	2286 (27.9%)	251 (30.3%)	56 (37.3%)	0.016
AMI	n (%)	991 (9.0%)	476 (10.9%)	246 (5.5%)	23 (7.5%)	<0.001	651 (7.9%)	78 (9.4%)	16 (10.7%)	0.175
CCI (0-24)	Median (IQR)	5 (3-8)	6(3-9)	5(2-7)	6(4-8)	<0.001	5(3-8)	6(4-9)	6(4-9)	<0.001
Van Elixhauser index	Median (IQR)	5 (0-14)	6(0-16)	3(0-12)	6(0-14)	<0.001	4(0-14)	5(0-15)	9(0-16)	<0.001
Statin use	n (%)	2823 (25.7%)	1240 (28.4%)	728(16.2%)	79(25.7%)	<0.001	1792(21.9%)	202(24.4%)	51(34%)	<0.001
Low		93(0.9%)	40(0.9%)	34(0.8%)	2(0.7%)		70(0.9%)	5(0.6%)	0(0%)	
Intermediate		723 (6.6%)	395(9.0%)	214(4.8%)	17(5.5%)	<0.001	545(6.7%)	71(8.6%)	10(6.7%)	<0.001
High		2007(18.2%)	805(18.4%)	480(10.7%)	60(19.5%)		1177(14.4%)	126(15.2%)	41(27.3%)	
DHP CCB	n (%)	2023 (18.4%)	858(19.6%)	644(14.3%)	60(19.5%)	<0.001	1379(16.8%)	144(17.4%)	35(23.3%)	0.105
Non DHP CCB	n (%)	628 (5.7%)	283(6.5%)	203(4.5%)	15(4.9%)	<0.001	429(5.2%)	57(6.9%)	14(9.3%)	0.015
ACE inhibitors	n (%)	1156 (10.5%)	466(10.7%)	354(7.9%)	20 (6.5%)	<0.001	730(8.9%)	88(10.6%)	21(14%)	0.031
Labs										
Albumin (g/dL)	Median (IQR)	3.8(3.5-4.0)	3.7(3.4-4)	3.9(3.6-4.1)	3.8(3.5-4.1)	<0.001	3.8(3.5-4.0)	3.8(3.6-4.1)	3.8(3.6-4.1)	0.014
AST (units/L)	Median (IQR)	22.6(18.8-28.7)	22.6(18.7-29)	22.5(19-28)	22.3(18.7-29)	0.119	22.3(18.7-28)	24(20-30.5)	24(19-33.2)	0.911
ALT (units/L)	Median (IQR)	21(16-29)	21(16-29)	21(16-29)	21(16-30)	0.251	20.8(16-28.0)	25.1(19-34.3)	28.6(20-4.1)	0.037
INR	Mean (SD)	1.1(1-1.2)	1.1(1-1.3)	1.1(1-1.2)	1(0.9-1.1)	<0.001	1.1(1-1.2)	1.1(1-1.4)	1(1-1.1)	0.032
Creatinine (mg/dL)	Median (IQR)	0.9(0.8-1.2)	1(0.8-1.3)	0.9(0.8-1.1)	0.9(0.8-1.2)	<0.001	0.9(0.8-1.2)	1(0.8-1.3)	1(0.8-1.6)	0.001
BUN (mg/dL)	Median (IQR)	14(10.4-19)	14.5(11-21)	13.2(10-17.8)	13.5(9.9-19)	<0.001	14(10.1-19)	15(11.3-21)	14(12-23.6)	<0.001
eGFR (mL/min/1.73m^2)	Median (IQR)	73(49.1-89.7)	70.8(44.3-89.7)	74(54.4-89.7)	70.7(54.5-89.8)	0.207	73.2(50.6-89.3)	71.8(47.1-89.9)	79.9(39.4-90)	0.878

AA, African American; ALT Alanine transaminase; AMI, History of Acute myocardial infarction, AST, Aspartate transaminase; BMI, body mass index; BUN – Blood Urea Nitrogen; CAD, coronary artery disease; CAD, Coronary artery disease; CCI, Charlson comorbidity index; CKD, chronic kidney disease (≥Stage 3); DHP CCB, dihydropyridine calcium channel blocker; non DHP CCB, non-dihydropyridine calcium channel blocker; DM, diabetes mellitus; eGFR, Estimated Glomerular filtration rate; INR – International normalized ratio, IQR, Interquartile range; HIV, human immunodeficiency virus; HTN, hypertension; IQR, interquartile range; PVD, peripheral vascular disease; SD, standard deviation, TC, Total Cholesterol; TG, Triglycerides;

Supplementary table 2: Lipid levels in each of the corresponding trajectories and tertiles of lipids during the 2 years antecedent to COVID-19 testing.

Type of Lipid	Tertiles	No. of patients	Lipid levels (mg/dL) Median (Range)	Trajectory	No. of patients	Lipid levels (mg/dL) Median (Range)
LDL-C	1st	3104	70 (7-87)	1st	5157	82(7-130)
	2nd	3044	103(87.3-119)	2nd	3709	132(82-215)
	3rd	3054	140(119.3-242)	3rd	189	202.5(155-242)
HDL-C	1st	3138	35(5-41.5)	1st	5687	40.5 (14-52)
	2nd	3099	47(41.6-54)	2nd	2971	60.7 (50.5-83.1)
	3rd	3087	64.5(54.3-148)	3rd	495	88(76.4-148)
TC	1st	3061	139(29-159)	1st	4370	148(51-194.5)
	2nd	3078	179(159.3-197)	2nd	4494	204(144.5-277)
	3rd	3039	222(197.5-636)	3rd	307	286(242.5-636)
TG	1st	3155	61(13-82)	1st	8187	97.0(13.0-711.5)
	2nd	3085	105.5(82.2-137)	2nd	828	278.4(189.5-729.0)
	3rd	3095	193(137-1491)	3rd	150	515.9(391.0-1453.5)

LDL-C, Low Density Lipoprotein Cholesterol; HDL-C, High Density Lipoprotein Cholesterol; TC, Total Cholesterol; TG, Triglycerides.

Supplementary table 3: Median (IQR) Values of lipid levels in the trajectories of each of the other lipid fractions during the 2 years antecedent to COVID-19 testing.

Characteristics	Total (N=11001)	LDL-C			p-value	HDL-C			p-value
		Trajectory 1	Trajectory 2	Trajectory 3		Trajectory 1	Trajectory 2	Trajectory 3	
HDL-C (mg/dL)	42(33-53)	42 (34-53)	45(35-57)	50(38.5-57)	0.009	40.5(34.5-46)	60.7(56-67)	88(83-96)	<0.001
LDL-C (mg/dL)	99(72.5-131)	79(62-100)	123 (95-150)	189(136-224.5)	<0.001	99(74.3-126)	108(86-132.7)	105(82.5-131.5)	<0.001
TC (mg/dL)	171(138-210)	149 (127-178)	200 (161.5-235)	265.5 (229-307.5)	<0.001	167.5(140-198)	190(166-215)	214(190-238)	<0.001
TG (mg/dL)	106.5 (75-163)	104 (75-164)	114 (81-168)	150(100-210)	0.317	122(83-184)	87.5(62-123.5)	65(49-92)	<0.001
**Characteristics**	**Total (N=11001)**	**TC**			**p-value**	**TG**			**p-value**
		**Trajectory 1**	**Trajectory 2**	**Trajectory 3**		**Trajectory 1**	**Trajectory 2**	**Trajectory 3**	
HDL-C (mg/dL)	42(33-53)	43(35-53)	51.5(42-63.2)	55(43-71)	<0.001	48.8(40-60)	38(32-45)	33(27-39)	<0.001
LDL-C (mg/dL)	99(72.5-131)	78.5(64-93)	125.5(110-142)	188(169.5-209)	<0.001	103(79-128)	102(72-134)	84(50.3-115)	<0.001
TC (mg/dL)	171(138-210)	148(130-162)	204(189-223.5)	286(269-303.7)	<0.001	176(148-205)	197.8(167.9-231.3)	213.4(173-247.5)	<0.001
TG (mg/dL)	106.5 (75-163)	92.2(63-138)	115(79-170)	177(114-283)	<0.001	97(67.5-137)	278.3(250-328.5)	515.9(457.8-602)	<0.001

TC, Total Cholesterol; TG, Triglycerides; HDL, High density lipoprotein cholesterol; LDL, low density lipoprotein cholesterol. TG, Triglycerides.

Supplementary Table 4A: Patient outcomes based on the tertiles of LDL-C and HDL-C during the 2 years antecedent to COVID-19 testing.

Patient outcomes	Unit	Total (N=11001)	LDL-C			p-value	HDL-C			p-value
			Tertile 1	Tertile 2	Tertile 3		Tertile 1	Tertile 2	Tertile 3	
Number of COVID-19 tests done	Median (IQR)	1(1-2)	2(1-3)	1(1-2)	1(1-2)	0.137	2(1-3)	1(1-2)	1(1-2)	0.082
Asymptomatic screening (%)	n (%)	5389 (54.7%)	1456(52.5%)	1470(54.4%)	1408 (52.9%)	0.304	1431(51.5%)	1442(52.7%)	1508(55.2%)	0.018
Number of patients testing positive at least once for COVID-19	n (%)	1340 (12.2%)	393 (12.7%)	390 (12.8%)	421 (13.8%)	0.367	464(14.8%)	409(13.2%)	349(11.3%)	<0.001
COVID-19 outcomes										
Admission for COVID-19 (%)	n (%)		107 (27.2%)	95 (24.4%)	86 (20.4%)	0.073	127(27.4%)	95(23.2%)	72(20.6%)	0.075
Duration of hospital admission (days)	Median (IQR)	6 (3-12)	6 (3-14)	6 (4-12)	4 (3-10)	0.381	6(3-12)	6(3-12)	5(3-8)	0.177
Severe COVID-19 (%) at admission	n (%)	86/366 (23.5%)	27/112 (24.1%)	25/98 (25.5%)	17/88 (19.3%)	0.580	37/130 (28.5%)	19/99(19.2%)	16/75(21.3%)	0.226
Severe COVID-19 (%) anytime during hospitalization	n (%)	106/366 (28.9%)	35/112 (31.3%)	29/98 (29.6%)	22/88 (25.0%)	0.614	45/130(34.6%)	25/99(25.3%)	19/75(25.3%)	0.209
Morality due to COVID-19 (%)	n (%)	59/366 (16.1%)	22/112(19.6%)	15/98(15.3%)	13/88 (14.8%)	0.587	24/130(18.5%)	15/99(15.2%)	12/75(16.0%)	0.785
Highest oxygen support required<br>- None<br>- Nasal cannula<br>- High flow oxygen<br>- NIV<br>- Intubation	n (%)	 <br>115/366 (31.4%)<br>143/366 (39.2%)<br>44/366 (12.1%)<br>13/366 (3.6%)<br>51/366 (13.9%)	 <br>34/112 (30.4%)<br>42/112 (37.5%)<br>11/112 (9.8%)<br>6/112 (5.4%)<br>19 /112 (16.7%)	 <br>34/98 (34.7%)<br>34/98 (34.7%)<br>13/98 (13.3%)<br>3/98 (3.1%)<br>14/98 (14.3%)	 <br>27/88(30.7%)<br>38/88 (43.2%)<br>10/88 (11.4%)<br>3/88 (3.4%)<br>10/88 (11.4%)	0.884	 <br>39/130 (30.0%)<br>45/130 (34.6%)<br>16/130 (12.3%)<br>8/130 (6.2%)<br>22/130 (16.9%)	 <br>33/99 (33.3%)<br>41/99 (41.4%)<br>9/99 (9.1%)<br>3/99 (3.0%)<br>13/99 (13.1%)	 <br>24/75 (32.0%)<br>30/75 (40.0%)<br>11/75 (14.7%)<br>2/75 (2.7%)<br>8/75 (10.7%)	0.709
ECMO	n (%)	1/366 (0.3%)	0 (0%)	1 (1.0%)	0/88 (0%)	0.361	0/130 (0%)	1/99 (1.0%)	0/75 (0%)	0.356
Vasopressor use	n (%)	36/366 (9.9%)	13/112 (11.7%)	10/98 (10.2%)	6/88 (6.8%)	0.505	19/130 (14.7%)	8/99 (8.1%)	3/75 (4.0%)	0.036
Renal replacement	n (%)	42/366 (11.5%)	18 (16.2%)	12 (12.2%)	7 (7.9%)	0.215	22/130 (17.1%)	10/99 (10.1%)	5/75 (6.7%)	0.068

COVID-19, Coronavirus disease-2019; ECMO, Extracorporeal Membrane oxygenation; IQR, interquartile range; LDL-C, low density lipoprotein cholesterol; HDL-C, high density lipoprotein cholesterol; NIV, Non-invasive ventilation

Supplementary Table 4B: Patient outcomes based on the tertiles of TC and TG during the 2 years antecedent to COVID-19 testing.

Patient outcomes	Unit	Total (N=11001)	TC			p-value	TG			p-value
			Tertile 1	Tertile 2	Tertile 3		Tertile 1	Tertile 2	Tertile 3	
Number of COVID-19 tests done	Median (IQR)	1(1-2)	2(1-3)	1(1-2)	1(1-2)	0.006	1(1-2)	1(1-3)	2(1-3)	0.518
Asymptomatic screening (%)	n (%)	5389 (54.7%)	1425(52.1%)	1474 (54.3%)	1435(53.4%)	0.265	1503(54.3%)	1450(52.5%)	1429(52.4%)	0.286
Number of patients testing positive at least once for COVID-19	n (%)	1340 (12.2%)	411(13.4%)	397(12.9%)	392(12.9%)	0.778	398(12.6%)	438(14.2%)	388(12.5%)	0.092
COVID-19 outcomes										
Admission for COVID-19 (%)	n (%)	366 (27.3%)	108(26.3%)	89(22.4%)	95(24.2%)	0.441	70(17.6%)	111(25.3%)	114(29.4%)	<0.001
Duration of hospital admission (days)	Median (IQR)	6 (3-12)	7(3-14)	5(3-12)	4(2-10)	0.142	6(3-12)	5(3-11)	5(3-13)	0.783
Severe COVID-19 (%) at admission	n (%)	86/366 (23.5%)	29/111(26.1%)	25/94(26.6%)	17/97(17.5%)	0.240	17/77(23.3%)	26/112(23.2%)	29/120(24.2%)	0.983
Severe COVID-19 (%) anytime during hospitalization	n (%)	106/366 (28.9%)	38/111(34.2%)	29/94(30.9%)	21/97(21.7%)	0.125	77/260(29.6%)	6/32(18.8%)	5/10(50%)	0.149
Morality due to COVID-19 (%)	n (%)	59/366 (16.1%)	22/111(19.8%)	14/94(14.9%)	14/97 (14.4%)	0.507	15/73(20.6%)	19/112(16.9%)	17/120(14.2%)	0.513
Highest oxygen support required<br>- None<br>- Nasal cannula<br>- High flow oxygen<br>- NIV<br>- Intubation	n (%)	 <br>115/366 (31.4%)<br>143/366 (39.2%)<br>44/366 (12.1%)<br>13/366 (3.6%)<br>51/366 (13.9%)	 <br>31/110 (27.9%)<br>41/110 (36.9%)<br>15/110 (13.5%)<br>5/110 (4.5%)<br>19/110 (17.1%)	 <br>32/94 (34.0%)<br>32/94 (64.0%)<br>10/94 (10.6%)<br>4/94 (4.3%)<br>16/94 (17.0%)	 <br>33/97 (34.0%)<br>42/97 (43.3%)<br>10/97 (10.3%)<br>4/97 (4.1%)<br>8/97 (8.3%)	0.631	 <br>19/73 (26.0%)<br>29/73 (39.7%)<br>10/73 (13.7%)<br>2/73 (2.7%)<br>13/73 (17.8%)	 <br>37/112 (33.0%)<br>43/112 (38.4%)<br>11/112 (9.8%)<br>4/112 (3.6%)<br>17/112 (15.2%)	 <br>41/120 (34.2%)<br>44/120 (36.7%)<br>15/120 (12.5%)<br>7/120 (5.8%)<br>13/120 (10.8%)	0.787
ECMO	n (%)	1/366 (0.3%)	0/110 (0%)	1/94 (1.1%)	0(0%)	0.331	0/72(0%)	0(0%)	1(0.8%)	0.463
Vasopressor use	n (%)	36/366 (9.9%)	16/110(14.6%)	6/94 (6.4%)	7/97 (7.2%)	0.089	5/72(6.9%)	11(9.8%)	14(11.7%)	0.569
Renal replacement	n (%)	42/366 (11.5%)	20/110 (18.2%)	6/94 (6.4%)	10/97 (10.3%)	0.029	6/72(8.3%)	16/112(14.3%)	15/120 (12.5%)	0.479

COVID-19, Coronavirus disease-2019; ECMO, Extracorporeal Membrane oxygenation; IQR, interquartile range; TC, Total Cholesterol; TG, Triglycerides; NIV, Non-invasive ventilation

Supplementary table 5A: Sensitivity analysis - Association of trajectories and tertiles of pre-testing lipid levels with the risk of testing positive for COVID-19 using mixed-effects Poisson regression analysis including all available test results.

Type of lipid	Trajectory	Unadjusted RR	Adjusted RR Model 1	Adjusted RR Model 2	Tertile	Unadjusted RR	Adjusted RR Model 1	Adjusted RR Model 2
LDL-C	Trajectory 1	Ref	Ref	Ref	Tertile 1	Ref	Ref	Ref
	Trajectory 2	1.18 [1.05-1.33]	1.09 [0.97–1.23]	1.08 [0.96-1.22]	Tertile 2	1.11 [0.96-1.28]	0.97 [0.84-1.12]	0.98 [0.85-1.13]
	Trajectory 3	1.13 [0.78-1.65]	1.04 [0.73-1.49]	1.02 [0.71-1.47]	Tertile 3	1.24 [1.08-1.42]	1.09 [0.95-1.25]	1.08 [0.94-1.25]
HDL-C	Trajectory 1	Ref	Ref	Ref	Tertile 1	Ref	Ref	Ref
	Trajectory 2	0.93 [0.82-1.06]	0.87 [0.76-0.98]	0.92 [0.81-1.05]	Tertile 2	0.98 [0.86-1.12]	0.87 [0.76-0.99]	0.91 [0.79-1.04]
	Trajectory 3	0.59 [0.43-0.81]	0.56 [0.40-0.77]	0.62 [0.48-0.86]	Tertile 3	0.87 [0.75-1.00]	0.76 [0.66-0.89]	0.84 [0.73-0.98]
TC	Trajectory 1	Ref	Ref	Ref	Tertile 1	Ref	Ref	Ref
	Trajectory 2	1.10 [0.98-1.23]	1.04[0.93-1.17]	1.05 [0.93-1.18]	Tertile 2	1.13 [0.98-1.30]	1.02 [0.89-1.17]	1.01 [0.88-1.16]
	Trajectory 3	0.92 [0.66-1.27]	0.91 [0.66-1.24]	0.89 [0.65-1.22]	Tertile 3	1.09 [0.94-1.25]	1.01 [0.87-1.17]	1.00 [0.87-1.16]
TG	Trajectory 1	Ref	Ref	Ref	Tertile 1	Ref	Ref	Ref
	Trajectory 2	1.11 [0.96-1.28]	0.91 [0.73-1.14]	0.85 [0.68-1.06]	Tertile 2	1.09 [0.96-1.26]	1.23 [1.08-1.41]	1.18 [1.03-1.35]
	Trajectory 3	1.24 [1.08-1.42]	1.03 [0.68-1.55]	0.95 [0.63-1.43]	Tertile 3	0.82 [0.79-1.06]	1.13 [0.98-1.30]	1.03 [0.89-1.18]

Adjusted Model 1: Age + Gender + Race + CCI + Statin intensity + DHPCCB + nDHPCCB + ACEI + Alcohol intake + Times tested
Adjusted Model 2: Model 1 + DM + HTN + HIV + BMI
DHPCCB, dihydropyridine calcium channel blocker; nDHPCCB, non-dihydropyridine calcium channel blocker; CCI, Charlson comorbidity index; DM, diabetes mellitus; TC, Total Cholesterol; TG, Triglycerides; HIV, human immunodeficiency virus; HTN, hypertension; IQR, interquartile range; HDL-C, High density lipoprotein cholesterol; LDL-C, low density lipoprotein cholesterol; Ref, Reference group; *RR, Relative risk of testing positive for COVID-19*

Supplementary table 5B: Sensitivity analysis - Association of mean antecedent lipid levels of each patient in the past 2 years (as continuous variables) with the risk of testing positive for COVID-19 using log-binomial regression

Type of lipid	Unadjusted RR	p-value	Adjusted RR Model 1	p-value	Adjusted RR Model 2	p-value
LDL-C	1.001 [0.999-1.003]	0.097	1.001 (0.999-1.002)	0.331	1.001 (0.999-1.002)	0.348
HDL-C	0.994 [0.990-0.997]	<0.001	0.993 (0.990-0.997)	<0.001	0.996 (0.992-0.999)	0.030
TC	0.999 [0.998-1.001]	0.688	0.999 (0.998-1.001)	0.871	0.999 (0.998-1.001)	0.868
TG	0.999 [0.999-1.001]	0.961	1.000 (0.999-1.001)	0.115	1.000 (0.999-1.001)	0.665

Adjusted Model 1: Age + Gender + Race + CCI + Statin intensity + DHPCCB + nDHPCCB + ACEI + Alcohol intake + Times tested
Adjusted Model 2: Model 1 + DM + HTN + HIV + BMI
DHPCCB, dihydropyridine calcium channel blocker; nDHPCCB, non-dihydropyridine calcium channel blocker; CCI, Charlson comorbidity index; DM, diabetes mellitus; TC, Total Cholesterol; TG, Triglycerides; HIV, human immunodeficiency virus; HTN, hypertension; IQR, interquartile range; HDL-C, High density lipoprotein cholesterol; LDL-C, low density lipoprotein cholesterol; Ref, Reference group; *RR, Relative risk of testing positive for COVID-19*

Supplementary table 5C: Sensitivity analysis - Association of trajectories and tertiles of pre-testing lipid levels with the risk of testing positive for COVID-19 using log-binomial regression after adjusting for the setting of COVID-19 testing

Type of lipid	Trajectory	Adjusted RR #	Tertile	Adjusted RR #
LDL-C	Trajectory 1	Ref	Tertile 1	Ref
	Trajectory 2	1.08 [0.98-1.20]	Tertile 2	0.99 [0.87-1.13]
	Trajectory 3	1.04 [0.75-1.45]	Tertile 3	1.08 [0.96-1.23]
HDL-C	Trajectory 1	Ref	Tertile 1	Ref
	Trajectory 2	0.91 [0.81-1.02]	Tertile 2	0.92 [0.81-1.04]
	Trajectory 3	0.62 [0.46-0.85]	Tertile 3	0.84 [0.73-0.96]
TC	Trajectory 1	Ref	Tertile 1	Ref
	Trajectory 2	1.04 [0.94-1.15]	Tertile 2	0.98 [0.86-1.11]
	Trajectory 3	0.95 [0.71-1.26]	Tertile 3	1.01 [0.89-1.14]
TG	Trajectory 1	Ref	Tertile 1	Ref
	Trajectory 2	0.82[0.52-1.21]	Tertile 2	1.23[1.04-1.46]
	Trajectory 3	1.01[0.42-1.62]	Tertile 3	1.04[0.86-1.24]

Model adjusted for: Age + Gender + Race + CCI + Statin intensity + DHPCCB + nDHPCCB + ACEI + Alcohol intake + Times tested + DM + HTN + HIV + + BMI + Setting of COVID-19 testing
DHPCCB, dihydropyridine calcium channel blocker; nDHPCCB, non-dihydropyridine calcium channel blocker; CCI, Charlson comorbidity index; DM, diabetes mellitus; TC, Total Cholesterol; TG, Triglycerides; HIV, human immunodeficiency virus; HTN, hypertension; HDL-C, High density lipoprotein cholesterol; LDL-C, low density lipoprotein cholesterol; Ref, Reference group; RR, Relative risk of testing positive for COVID-19; TC, Total cholesterol; TG, Triglycerides
Setting of COVID-19 testing: Either asymptomatic screening or symptomatic testing

Supplementary table 5D: Association of tertiles of pre-testing non-HDL levels with the risk of testing positive for COVID-19 using log-binomial regression

Type of lipid	Tertile	Unadjusted RR	Adjusted RR Model 1	Adjusted RR Model 2
Non-HDL-C	Tertile 1	Ref	Ref	Ref
	Tertile 2	1.07 [0.94-1.22]	1.07 [0.94-1.21]	1.04 [0.91-1.18]
	Tertile 3	1.07 [0.94-1.22]	1.09 [0.95-1.23]	1.05 [0.92-1.19]

Adjusted Model 1: Age + Gender + Race + CCI + Statin intensity + DHPCCB + nDHPCCB + ACEI + Alcohol intake + Times tested
Adjusted Model 2: Model 1 + DM + HTN + HIV + BMI + Vaccination
DHPCCB, dihydropyridine calcium channel blocker; nDHPCCB, non-dihydropyridine calcium channel blocker; CCI, Charlson comorbidity index; DM, diabetes mellitus; TC, Total Cholesterol; TG, Triglycerides; HIV, human immunodeficiency virus; HTN, hypertension; IQR, interquartile range; HDL-C, High density lipoprotein cholesterol; LDL-C, low density lipoprotein cholesterol; Ref, Reference group; RR, Relative risk of COVID-19 positivity

Supplementary table 6: Association of trajectories and tertiles of antecedent lipid levels with the risk of admission for COVID-19 using log-binomial regression

Type of lipid	Trajectory	Unadjusted RR	Adjusted RR Model 1	Adjusted RR Model 2	Tertile	Unadjusted RR	Adjusted RR Model 1	Adjusted RR Model 2
LDL-C	Trajectory 1	Ref	Ref	Ref	Tertile 1	Ref	Ref	Ref
	Trajectory 2	0.76[0.61-0.94]	.097 [0.68-1.37]	0.95[0.67-1.35]	Tertile 2	0.89[0.71-1.14]	1.33[0.88-2.03]	1.33[0.88-2.02]
	Trajectory 3	0.93[0.48-1.79]	0.65 [0.21-2.06]	0.65[0.21-2.07]	Tertile 3	0.75[0.59-0.96]	1.16[0.77-1.77]	1.16[0.77-1.77]
HDL-C	Trajectory 1	Ref	Ref	Ref	Tertile 1	Ref	Ref	Ref
	Trajectory 2	0.78 [0.62-0.99]	0.86 [0.58-1.28]	0.83[0.55-1.24]	Tertile 2	0.89[0.67-1.07]	1.14[0.77-1.7]	1.14[0.77-1.70]
	Trajectory 3	0.88 [0.49-1.59]	1.24[0.48-3.17]	1.13[0.43-2.93]	Tertile 3	0.75[0.58-0.97]	1.04[0.66-1.63]	1.00[0.63-1.59]
TC	Trajectory 1	Ref	Ref	Ref	Tertile 1	Ref	Ref	Ref
	Trajectory 2	0.82 [0.67-1.01]	1.04[0.74-1.47]	1.03[0.73-1.45]	Tertile 2	0.98[0.88-1.09]	1.00[0.90-1.12]	1.00[0.90-1.12]
	Trajectory 3	0.96 [0.55-1.67]	1.02[0.39-2.67]	0.99[0.38-2.59]	Tertile 3	0.96[0.71-1.30]	0.95[0.71-1.28]	0.94[0.70-1.27]
TG	Trajectory 1	Ref	Ref	Ref	Tertile 1	Ref	Ref	Ref
	Trajectory 2	1.47[1.08-2.00]	1.07[0.61-1.88]	1.07[0.61-1.90]	Tertile 2	1.44[1.10-1.88]	1.02[0.66-1.57]	1.03[0.67-1.59]
	Trajectory 3	1.87[1.16-3.03]	1.22[0.40-3.72]	1.25[0.41-3.80]	Tertile 3	1.67[1.28-2.17]	1.21[0.78-1.87]	1.25[0.81-1.95]

Adjusted Model 1: Age + Gender + Race + CCI + Statin intensity + DHPCCB + nDHPCCB + ACEI + Alcohol intake
Adjusted Model 2: Model 1 + DM + HTN + HIV + BMI
DHPCCB, dihydropyridine calcium channel blocker; nDHPCCB, non-dihydropyridine calcium channel blocker; CCI, Charlson comorbidity index; DM, diabetes mellitus; TC, Total Cholesterol; TG, Triglycerides; HIV, human immunodeficiency virus; HTN, hypertension; IQR, interquartile range; HDL, High density lipoprotein cholesterol; LDL, low density lipoprotein cholesterol; RR, Relative risk of admission for COVID-19

Supplementary table 7: Association of trajectories and tertiles of antecedent lipid levels with the risk of severe COVID-19 among patients admitted for COVID-19 using log-binomial regression

Type of lipid	Trajectory	Unadjusted RR	Adjusted RR Model 1	Adjusted RR Model 2	Tertile	Unadjusted RR	Adjusted RR Model 1	Adjusted RR Model 2
LDL-C	Trajectory 1	Ref	Ref	Ref	Tertile 1	Ref	Ref	Ref
	Trajectory 2	0.73[0.49-1.09]	0.69[0.39-1.25]	0.69[0.38-1.26]	Tertile 2	0.96[0.64-1.45]	1.02[0.54-1.92]	0.99[0.53-1.89]
	Trajectory 3	0.43[0.07-2.66]	0.36[0.04-3.33]	0.33[0.04-3.09]	Tertile 3	0.81[0.51-1.27]	0.94[0.47-1.86]	0.93[0.46-1.86]
HDL-C	Trajectory 1	Ref	Ref	Ref	Tertile 1	Ref	Ref	Ref
	Trajectory 2	0.92[0.61-1.39]	0.89[0.46-1.72]	0.89[0.45-1.75]	Tertile 2	0.76[0.50-1.45]	0.62[0.33-1.19]	0.62[0.32-1.19]
	Trajectory 3	0.37[0.06-2.37]	0.31[0.04-2.71]	0.29[0.03-2.69]	Tertile 3	0.76[0.48-1.20]	0.61[0.29-1.27]	0.59[0.27-1.28]
TC	Trajectory 1	Ref	Ref	Ref	Tertile 1	Ref	Ref	Ref
	Trajectory 2	0.73[0.51-1.06]	0.77[0.44-1.34]	0.79[0.45-1.40]	Tertile 2	0.98[0.88-1.09]	1.00[0.90-1.12]	1.00[0.90-1.12]
	Trajectory 3	0.60[0.17-2.09]	0.49[0.09-2.59]	0.46[0.09-2.47]	Tertile 3	0.96[0.71-1.30]	0.95[0.71-1.28]	0.94[0.70-1.27]
TG	Trajectory 1	Ref	Ref	Ref	Tertile 1	Ref	Ref	Ref
	Trajectory 2	0.63[0.31-1.33]	0.59[0.22-1.60]	0.58[0.51-1.61]	Tertile 2	0.88[0.56-1.37]	0.73[0.36-1.47]	0.74[0.36-1.49]
	Trajectory 3	1.69[0.88-3.23]	2.24[0.53-9.42]	2.68[0.60-11.92]	Tertile 3	0.88[0.57-1.37]	0.85[0.42-1.72]	0.88[0.43-1.80]

Adjusted Model 1: Age + Gender + Race + CCI + Statin intensity + DHPCCB + nDHPCCB + ACEI + Alcohol intake
Adjusted Model 2: Model 1 + DM + HTN + HIV + BMI
DHPCCB, dihydropyridine calcium channel blocker; nDHPCCB, non-dihydropyridine calcium channel blocker; CCI, Charlson comorbidity index; DM, diabetes mellitus; TC, Total Cholesterol; TG, Triglycerides; HIV, human immunodeficiency virus; HTN, hypertension; IQR, interquartile range; HDL, High density lipoprotein cholesterol; LDL, low density lipoprotein cholesterol; RR, Relative risk of severe COVID-19

Supplementary table 8: Association of trajectories and tertiles of antecedent lipid levels with the hazard for in-hospital mortality among patients admitted for COVID-19 using cox-proportional hazards model

Type of lipid	Trajectory	Unadjusted HR	Adjusted HR Model 1	Adjusted HR Model 2	Tertile	Unadjusted HR	Adjusted HR Model 1	Adjusted HR Model 2
LDL-C	Trajectory 1	Ref	Ref	Ref	Tertile 1	Ref	Ref	Ref
	Trajectory 2	1.06[0.55-2.03]	1.24[0.58-2.66]	1.42[0.65-3.09]	Tertile 2	0.77[0.37-1.61]	0.56[0.24-1.33]	0.55[0.23-1.29]
	Trajectory 3	-	-	-	Tertile 3	1.02[0.48-2.14]	0.85[0.36-2.03]	0.99[0.41-2.43]
HDL-C	Trajectory 1	Ref	Ref	Ref	Tertile 1	Ref	Ref	Ref
	Trajectory 2	0.95[0.45-1.99]	0.84[0.38-1.90]	0.80[0.35-1.82]	Tertile 2	0.87[0.43-1.76]	0.69[0.32-1.48]	0.68[0.32-1.46]
	Trajectory 3	5.94[0.75-47.06]	4.86[0.49-48.15]	3.64[0.35-37.5]	Tertile 3	1.35[0.63-2.89]	1.05[0.44-2.48]	0.98[0.41-2.38]
TC	Trajectory 1	Ref	Ref	Ref	Tertile 1	Ref	Ref	Ref
	Trajectory 2	0.93[0.49-1.74]	0.81[0.40-1.65]	0.85[0.42-1.75]	Tertile 2	0.98[0.88-1.09]	1.00[0.90-1.12]	1.00[0.90-1.12]
	Trajectory 3	-	-	-	Tertile 3	0.96[0.71-1.30]	0.95[0.71-1.28]	0.94[0.70-1.27]
TG	Trajectory 1	Ref	Ref	Ref	Tertile 1	Ref	Ref	Ref
	Trajectory 2	0.19[0.03-1.37]	0.21[0.03-1.60]	0.24[0.03-1.86]	Tertile 2	0.91[0.45-1.86]	0.96[0.42-2.18]	0.99[0.43-2.29]
	Trajectory 3	0.51[0.07-3.69]	0.24[0.03-1.91]	0.22[0.03-1.78]	Tertile 3	0.56[0.26-1.25]	0.45[0.16-1.29]	0.49[0.17-1.42]

Adjusted Model 1: Age + Gender + Race + CCI + Statin intensity + DHPCCB + nDHPCCB + ACEI + Alcohol intake
Adjusted Model 2: Model 1 + DM + HTN + HIV + BMI
DHPCCB, dihydropyridine calcium channel blocker; nDHPCCB, non-dihydropyridine calcium channel blocker; CCI, Charlson comorbidity index; DM, diabetes mellitus; TC, Total Cholesterol; TG, Triglycerides; HIV, human immunodeficiency virus; HTN, hypertension; IQR, interquartile range; HDL, High density lipoprotein cholesterol; LDL, low density lipoprotein cholesterol; RR, Relative risk of admission for COVID-19
*Person-time at risk of mortality, for each of the lipid trajectories and tertiles, was calculated from the time of hospital admission to 40 days, or discharge or transfer to another hospital or administrative censoring.

Supplementary table 9: Association of antecedent lipid levels with CRP levels following COVID-19 infection using mixed-effects linear regression analysis

Type of Lipid	Trajectory	CRP		Adjusted Model 1		Adjusted Model 2		Tertile	Unadjusted		Adjusted Model 1		Adjusted Model 2	
		Univariable B (SE)	p-value	Multvariable B (SE)	p-value	Multvariable B (SE)	p-value		Univariable B (SE)	p-value	Multvariable B (SE)	p-value	Multvariable B (SE)	p-value
LDL-C	Trajectory 1	Ref	-	Ref	-	Ref	-	Tertile 1	Ref	-	Ref		Ref	-
	Trajectory 2	-14.7(10.6)	0.168	-12.5(10.7)	0.244	-12.2(10.6)	0.254	Tertile 2	2.40(11.4)	0.832	4.73(11.28)	0.675	4.29(11.29)	0.704
	Trajectory 3	3.7(43.4)	0.932	6.45(43.1)	0.881	6.1(43.3)	0.889	Tertile 3	-16.7(12.2)	0.172	-13.4(12.0)	0.266	13.97(12.1)	0.247
HDL-C	Trajectory 1	Ref	-	Ref	-	Ref	-	Tertile 1	Ref	-	Ref	-	Ref	-
	Trajectory 2	-31.7(10.7)	0.003	-25.7(10.7)	0.017	-25.5(10.8)	0.018	Tertile 2	-21.22(11.7)	0.070	-14.58(11.7)	0.213	-14.6(11.7)	0.209
	Trajectory 3	-2.9(45.2)	0.948	12.1(44.8)	0.787	10.8(44.9)	0.810	Tertile 3	-34.43(12.5)	0.006	-25.2(-12.7)	0.049	-25.1(12.6)	0.048
TC	Trajectory 1	Ref	-	Ref	-	Ref	-	Tertile 1	Ref		Ref			-
	Trajectory 2	-13.7(10.3)	0.183	-11.8(10.2)	0.249	-11.3(10.2)	0.270	Tertile 2	-11.9(12.6)	0.346	-9.6(12.5)	0.444	-9.06(12.5)	0.468
	Trajectory 3	-5.3(39.5)	0.893	2.1(40.0)	0.959	0.9(40.0)	0.980	Tertile 3	-30.3(11.7)	0.010	-26.2(11.9)	0.030	-25.7(11.9)	0.032
TG	Trajectory 1	Ref	-	Ref	-	Ref	-	Tertile 1	Ref		Ref		Ref	-
	Trajectory 2	28.2(16.6)	0.090	22.9(16.6)	0.170	22.5(16.6)	0.175	Tertile 2	-1.95(13.3)	0.882	-7.7(13.0)	0.555	-7.4(13.2)	0.573
	Trajectory 3	-38.2(29.0)	0.189	-44.5(29.2)	0.128	-45.6(29.1)	0.119	Tertile 3	-10.1(12.9)	0.432	-16.1(12.9)	0.211	-15.8(12.9)	0.225

Adjusted Model 1: Age + Gender + Race + CCI + Statin intensity + DHPCCB + nDHPCCB + ACEI + Alcohol intake + Timestested
Adjusted Model 2: Model 1 + DM + HTN + HIV + BMI
B, linear regression co-efficient; SE – Standard error

Supplementary table 10: Association of antecedent lipid levels with ESR, Procalcitonin, IL-6 levels following COVID-19 infection using mixed-effects linear regression analysis

Type of Lipid	Trajectory (N=3690)	ESR		Procalcitonin		IL-6		Tertile	ESR		Procalcitonin		IL-6	
		Multvariable B (SE)	p-value	Multvariable B (SE)	p-value	Multvariable B (SE)	p-value		Multvariable B (SE)	p-value	Multvariable B (SE)	p-value	Multvariable B (SE)	p-value
LDL-C	Trajectory 1	Ref	-	Ref	-	Ref	-	Tertile 1	Ref	-	Ref	-	Ref	-
	Trajectory 2	-2.28(6.18)	0.712	-0.39(1.16)	0.735	131.3 (124.1)	0.290	Tertile 2	2.97 (7.19)	0.679	0.83(1.22)	0.497	6.48)198.8)	0.974
	Trajectory 3	-3.24(14.9)	0.828	-0.34(4.52)	0.940	121.8 (483.8)	0.801	Tertile 3	-2.97 (6.97)	0.670	-0.45(1.42)	0.918	17.09 (180.3)	0.825
HDL-C	Trajectory 1	Ref	-	Ref	-	Ref	-	Tertile 1	Ref	-	Ref	-	Ref	-
	Trajectory 2	-4.99(6.09)	0.413	0.23(1.08)	0.832	15.8(185.9)	0.933	Tertile 2	-1.17(7.06)	0.868	-0.43(1.27)	0.738	21.5 (171.6)	0.900
	Trajectory 3	-7.35(21.0)	0.727	0.49(4.03)	0.903	17.3 (471.4)	0.971	Tertile 3	2.34(7.17)	0.745	0.33(1.20)	0.785	43.8 (197.6)	0.825
TC	Trajectory 1	Ref	-	Ref	-	Ref	-	Tertile 1	Ref	-	Ref	-	Ref	-
	Trajectory 2	-2.71(5.68)	0.634	-0.21(1.14)	0.856	17.2(146.2)	0.906	Tertile 2	-1.71(4.28)	0.712	-0.21(1.14)	0.721	11.1 (112.2)	0.651
	Trajectory 3	-1.34(18.3)	0.942	0.21(3.87)	0.957	11.4(509.4)	0.982	Tertile 3	-1.40(15.2)	0.821	0.21(3.87)	0.815	16.5 (426.2)	0.699
TG	Trajectory 1	Ref	-	Ref	-	Ref	-	Tertile 1	Ref	-	Ref	-	Ref	-
	Trajectory 2	1.05 (9.13)	0.908	0.99 (1.31)	0.448	36 (237.6)	0.876	Tertile 2	0.24(6.82)	0.972	-0.08(1.28)	0.952	8.99(193.1)	0.963
	Trajectory 3	5.31 (27.8)	0.849	-8.31 (4.70)	0.077	-166.6 (521.1)	0.749	Tertile 3	-2.50(6.94)	0.720	-0.03(1.29)	0.985	-50.7 (207.2)	0.807

Multivariable Model: Adjusted for Age + Gender + Race + CCI + Statin intensity + DHPCCB + nDHPCCB + ACEI + Alcohol intake + DM + HTN + HIV +BMI
B, linear regression co-efficient; SE – Standard error
Multiple imputation using chained equations were used for the missing data. ESR: Erythrocyte sedimentation rate, IL6: Interleukin 6.
