# Supplementary material for: HDL cholesterol levels and susceptibility to COVID-19
Source: eBioMedicine. 2022 Jul 15;82:104166. doi: 10.1016/j.ebiom.2022.104166 (PMC9284176; doi:10.1016/j.ebiom.2022.104166)
Supplement: Supplementary file 2 [file mmc2.pdf]

UAMS
UNIVERSITY OF ARKANSAS
FOR MEDICAL SCIENCES
Institutional Review Board
4301 West Markham, #636
Little Rock, AR 72205-7199
501-686-5667
501-686-7265 (fax)
http://irb.uams.edu/

FWA00001119

10/15/2021

PI Name: Mehta, Jawahar
PI Department: COM Internal Med Card Acute Cardiac Care
Number: 263352
Project Title: Association of prior lipid levels with the development and prognosis of COVID-19 infection

NOT HUMAN SUBJECT RESEARCH DETERMINATION

The Institutional Review Board Director or Designee reviewed your material and determined that this project is NOT human subject research as defined in 45 CFR 46.102, and therefore it does not fall under the jurisdiction of the IRB review process.

Committee Notes/Comments:

- Per additional information provided, this project does not meet the definition of human subject research due to study staff receiving de-identified data from a third-party. IRB oversight is not needed.

Please keep the IRB advised of any changes that may require the project to be re-classified as human subject research.

If you have any questions, please contact an IRB administrator at 501-686-5667.
Click here to access study.

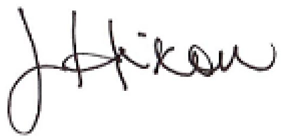

Jennifer Hixon
IRB Program Manager
